# Cardiolipin preserves T_reg_ metabolic fitness and immune homeostasis in the gut

**DOI:** 10.1038/s42255-026-01533-9

**Published:** 2026-05-18

**Authors:** Annamaria Regina, Francesca Solagna, Malkon Sanchez Estrada, Maaike M. E. Jacobs, Daniel Martinez-Martinez, Theodoros Georgomanolis, Irma Alibashikj, Sara Gjurgji, Claire Pearson, Dehui Chang, Chrysanthi Moschandrea, Ana Sagrera Aparisi, Elena Crespo, Jessica Buechel, Farina Schneider, Lea Trojahn, Paulina Pfelzer, Milica Popovic, Elena Potenza, Agnieszka M. Kabat, Carien Niessen, Borko Amulic, Niloufar Safinia, Sara Cogliati, David E. Sanin, Matteo Villa, Edward J. Pearce, Christian Frezza, Erika L. Pearce, Filipe Cabreiro, Fiona Powrie, Mauro Corrado

**Affiliations:** 1 Institute for Genetics, Faculty of Mathematics and Natural Sciences, University of Cologne, Cologne, Germany; 2 Cologne Excellence Cluster on Aging and Aging-associated Diseases (CECAD), University of Cologne, Cologne, Germany; 3 Center for Molecular Medicine Cologne (CMMC), University of Cologne, Cologne, Germany; 4 Max Planck Institute for Immunobiology and Epigenetics, Freiburg im Breisgau, Germany; 5 Institute of Clinical Sciences, Imperial College London, London, UK; 6 Institute for Metabolomics in Ageing, Faculty of Medicine and University Hospital Cologne, University of Cologne, Cologne, Germany; 7 Kennedy Institute of Rheumatology, NDORMS, University of Oxford, Oxford, UK; 8 Centro de Biologia Molecular Severo Ochoa (CBM), CSIC-UAM, and Institute for Molecular Biology-IUBM, Universidad Autónoma de Madrid, Madrid, Spain; 9 Roger Williams Institute of Liver Studies, School of Immunology & Microbial Sciences, Faculty of Life Sciences and Medicine, King’s College London University, and King’s College Hospital, London, UK; 10 Department Cell Biology of the Skin, Faculty of Medicine and University Hospital Cologne, University of Cologne, Cologne, Germany; 11 Institute for Mitochondrial Diseases in Ageing, Faculty of Medicine and University Hospital Cologne, University of Cologne, Cologne, Germany; 12 University of Bristol, Bristol, UK; 13 Department of Oncology, Bloomberg-Kimmel Institute for Cancer Immunotherapy, Johns Hopkins University School of Medicine, Baltimore, MD USA; 14 Division of Rheumatology and Immunology, Department of Internal Medicine, Medical University of Graz, Graz, Austria

**Keywords:** Inflammation, Immune tolerance, Stress signalling, Mucosal immunology

## Abstract

Loss of host–microbiota balance promotes gut inflammation, colitis and inflammatory bowel disease. Yet, whether host or microbial factors are the critical driver of the pathology remains unclear. Here, we investigate how cardiolipin maintains metabolic fitness of regulatory T (T_reg_) cells to preserve gut–immune homeostasis. We discover that deleting the cardiolipin-synthesizing enzyme protein tyrosine phosphatase mitochondrial 1 (PTPMT1) in T cells predisposes mice to colitis due to impaired T_reg_ cell function in the absence of dysbiosis. Subsequent pathobiont infections accelerate the progression and severity of gut inflammation. Mechanistically, the absence of cardiolipin impairs T_reg_ cell metabolic fitness and triggers a maladaptive integrated stress response, which can be reversed pharmacologically or genetically, restoring gut homeostasis and extending lifespan in PTPMT1 ΔT mice. Barth syndrome, a genetic disorder marked by severe cardiolipin deficiency, also exhibits gastrointestinal symptoms and inflammation associated with helper T cell imbalance and an active integrated stress response signature. Overall, these results suggest that a cardiolipin-mediated mitonuclear axis in T cells preserves gut–immune homeostasis and dictates outcome in pathobiont infections.

## Abstract (web summary)

This study describes that cardiolipin deficiency-induced mitochondrial dysfunction in T_reg_ cells impairs gut–immune tolerance.

## Main

The intestinal immune system must balance tolerance to food antigens and commensal microbiota while protecting from invading pathogens^1^. Loss of the host–microbiota balance triggers gut inflammation, colitis and inflammatory bowel disease (IBD)^2^. This makes the intestine a unique microenvironment to study immune cell function in response to the challenge represented by different microbial communities or by specific micro-organisms^3^. Of note, both microbial and genetic (immune) factors contribute to the pathogenesis of inflammatory conditions in the gut^4–8^. The presence of specific micro-organisms or dysbiosis is associated with development of colitis^4–6^. At the same time, genetic studies have identified multiple genetic loci associated with higher IBD risks^7,8^. Nevertheless, whether genetic factors are sufficient to trigger colitis or which factor primarily drives the pathology is not clear.

Pathobionts, including members of the *Helicobacter* family (*Helicobacter pylori*, *Helicobacter typhlonius*, *Helicobacter hepaticus*), establish lifelong infections in humans and mice if left untreated^9–11^. Yet, only a subset of infected individuals develops symptomatic infections, and not all mouse strains develop a pathogenic T cell-mediated colitis thanks to the production of interleukin (IL)-10 by the lamina propria-resident T_reg_ cells in the individuals and mice that successfully establish tolerance^12–16^. T_reg_ cells, a specialized subset of CD4^+^ T cells characterized by the expression of the transcription factor Forkhead box P3 (FOXP3), are gatekeepers of tissue homeostasis^17–19^. They suppress excessive or inappropriate immune activation, thereby preventing autoimmunity and tissue damage during infection and chronic inflammation and establishing immune tolerance^20,21^. T_reg_ cells share a common tissue-residency programme^22^. However, they show tissue-specific adaptation trajectories especially at barrier sites where the tissue microenvironment imposes tissue-specific metabolic pressures^23^.

Mitochondrial metabolism and fitness are crucial cell-autonomous checkpoints for T_reg_ cell immunosuppressive function^24–26^. They control T cell activation and differentiation into specific helper T cell subsets as well as their ability to establish long-term T cell immune memory^27–29^. Interfering with mitochondrial or other critical T cell functions can accelerate or cause tissue and systemic dysfunctions^30–32^. Yet, the role of mitochondrial metabolism in regulating immune tolerance is uncertain. Interestingly, individuals affected by mitochondrial deficiencies, part of the broader category of inborn errors of metabolism (IEMs), often present with gastrointestinal symptoms in addition to the more common neurological or cardiac ones^33,34^. Despite this, whether mitochondrial deficiencies might be risk factors or driving agents for gut inflammation to manifest is not known.

Cardiolipin is an anionic tetra-acyl chained glycerophospholipid, synthesized exclusively and localized mainly in the inner mitochondrial membrane^35^. It interacts with respiratory chain complexes to improve electron transfer efficiency and to reduce reactive oxygen species generation^36^. Moreover, it modulates multiple mitochondrial functions including stability of mitoribosomes and protein import into mitochondria^37,38^. Cardiolipin deficiency has been linked to neurodegeneration, heart failure and diabetes, although in most of these cases not as the driver of the condition^38^. Instead, Barth syndrome is caused by cardiolipin deficiency due to mutations in the *TAFAZZIN* gene, which encodes an acyl-transferase necessary to generate mature cardiolipin after its remodelling^39–41^. At the immune level, cardiolipin deficiency in Barth syndrome is linked to neutropenia, T cell alterations and susceptibility to infections^42,43^. Interestingly, some clinical reports suggest the presence of inflammation and gastrointestinal symptoms in these patients^44,45^. Yet, the responsible mechanism for these inflammatory symptoms has not been identified.

In response to diverse stress stimuli, including metabolic ones, eukaryotic cells activate a common adaptive pathway, named the integrated stress response (ISR)^46^. ISR inhibits protein synthesis globally but enhances the translation of a subset of genes to promote cellular recovery, including the transcription factors ATF4 and CHOP^46^. While generally a pro-survival programme, ISR can promote loss of cellular identity and cell death following severe or chronic stress^47–49^. Mitochondrial dysfunctions can activate ISR. However, little is known about their cross-talk in the context of immune tolerance regulation and cardiolipin deficiency.

We previously showed that PTPMT1 ΔT mice, lacking the enzyme required for cardiolipin synthesis in T cells, are characterized by lymphopenia and immune deficiency following an immune challenge with intravenous *Listeria monocytogenes* infection^43^. Here, using the PTPMT1 ΔT mouse model, we show that cardiolipin deficiency in T cells activates maladaptive ISR signalling, sensitizing mice to pathobiont infections and shifting the balance from tolerance to inflammation in a T_reg_ cell-dependent manner. In summary, we have identified and characterized a new and therapeutically targetable immunometabolic mechanism by which host mitochondrial dysfunctions impair gut–immune tolerance.

## Results

### Gut inflammation and reduced lifespan are hallmarks of PTPMT1 ΔT mice

**Fig. 1:**
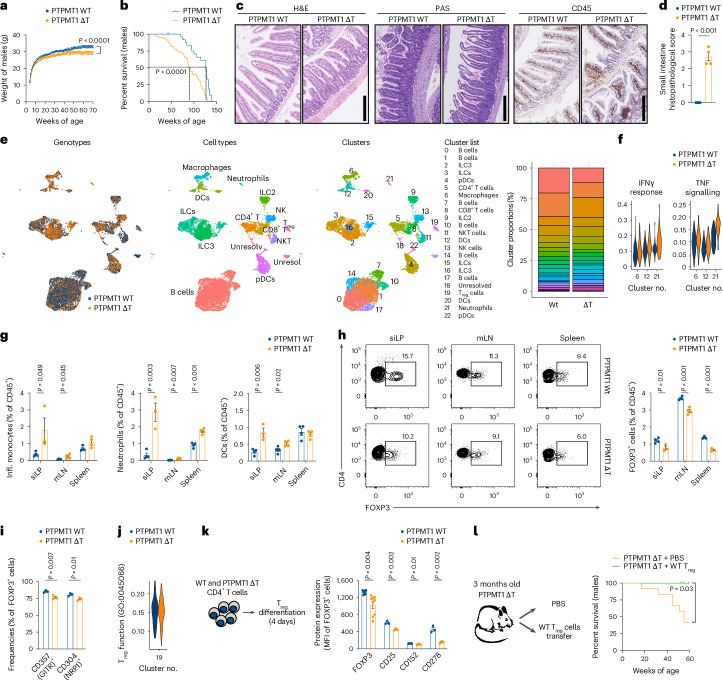
Helper T cell imbalance drives gut inflammation in PTPMT1 ΔT mice. **a**, Body weight curve of PTPMT1 WT (*N* = 30) and PTPMT1 ΔT (*N* = 57) male mice. **b**, Kaplan–Meier survival curve of PTPMT1 WT (*N* = 30) and PTPMT1 ΔT (*N* = 57) male mice. **c**, Representative histological images of small intestine sections from 12-week-old PTPMT1 WT and PTPMT1 ΔT mice stained with H&E, PAS and immunostained for CD45. Scale bars, 500 μm. **d**, Histopathology score of small intestines from PTPMT1 WT (*N* = 4) and PTPMT1 ΔT (*N* = 4) mice. **e**, Uniform manifold approximation and projection (UMAP) visualization of scRNA-seq data showing the distribution of the clusters and cell type identified in the small intestines from PTPMT1 WT and PTPMT1 ΔT mice. Cell numbers were normalized to 100% and the fractional contribution of every cluster is indicated. **f**, IFNγ and TNF signalling scores were calculated on the indicated clusters from scRNA-seq datasets. **g**, Frequency of infiltrating monocytes, neutrophils and dendritic cells (DCs) in the siLP (PTPMT1 WT *N* = 4 and PTPMT1 ΔT *N* = 3), mLNs (PTPMT1 WT *N* = 4 and PTPMT1 ΔT *N* = 4) and spleen (PTPMT1 WT *N* = 4 and PTPMT1 ΔT *N* = 4). **h**, Representative flow cytometry plots (left) and frequencies (right) of CD4^+^ FOXP3^+^ cells from siLP (PTPMT1 WT *N* = 4 and PTPMT1 ΔT *N* = 3), mLNs (PTPMT1 WT *N* = 4 and PTPMT1 ΔT *N* = 4) and spleen (PTPMT1 WT *N* = 3 and PTPMT1 ΔT *N* = 3). **i**, GITR and NRP1 expression levels in splenic CD4^+^ FOXP3^+^ cells isolated from PTPMT1 WT and PTPMT1 ΔT mice. **j**, T_reg_ immunosuppressive function score calculated from the T_reg_ cluster in the scRNA-seq dataset. **k**, FOXP3, CD25, CD152 (CTLA-4) and CD278 (ICOS) expression levels in PTPMT1 WT (*N* = 9 for FOXP3, *N* = 3 for CD25, CD152 and CD278) and PTPMT1 ΔT (*N* = 9 for FOXP3, *N* = 3 for CD25, CD152 and CD278) CD4^+^ cells after 4 days in T_reg_ differentiating culture condition. **l**, Kaplan–Meier survival curve of PTPMT1 ΔT mice injected with PBS (*N* = 12) or WT T_reg_ cells (*N* = 9). Data are shown as the mean ± s.e.m. of three independent experiments unless indicated. Statistical comparisons between two groups were calculated by using an unpaired two-tailed Student’s *t*-test. Log-rank (Mantel–Cox) test was performed to compare survival curves in **b** and **l**. Exact *P* values are indicated. NK, natural killer cells; ILC, innate lymphoid cells; pDC, plasmacytoid dendritic cells. Source data

**Extended Data Fig. 1:**
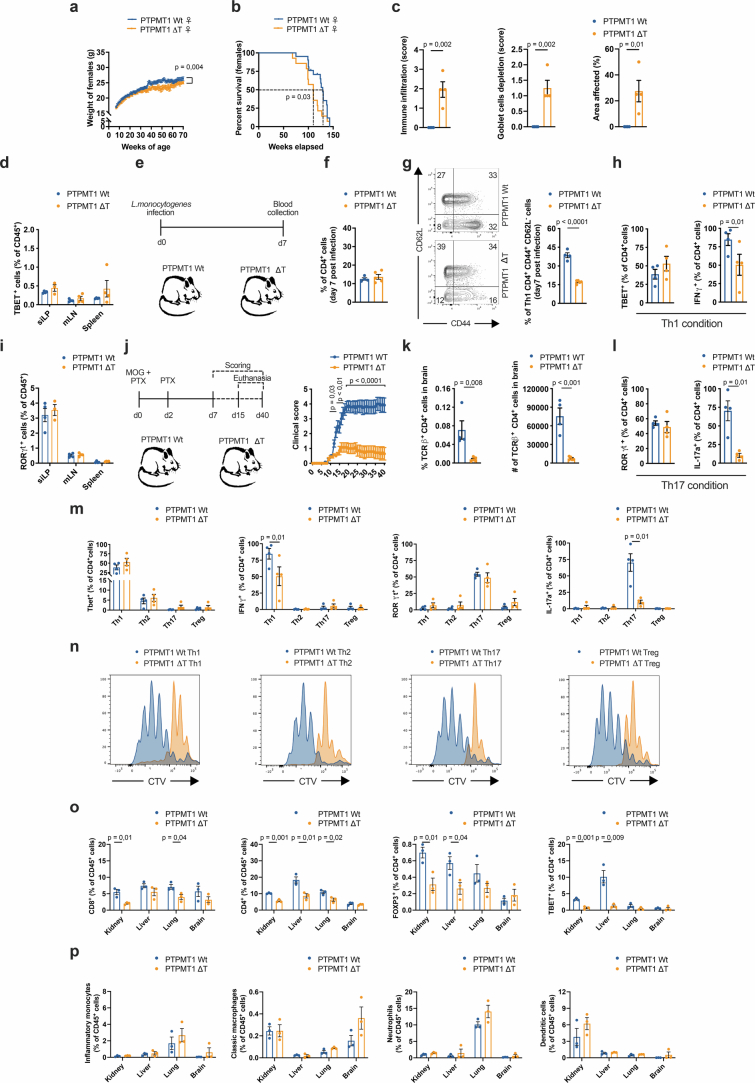
PTPMT1 loss results in CD4^+^ helper T cell subset imbalance. (**a**) Body weight curves of PTPMT1 Wt (N=21) and PTPMT1 ΔT (N=9) female mice. (**b**) Kaplan-Meier survival curves of PTPMT1 Wt (N=21) and PTPMT1 ΔT (N=9) female mice. (**c**) Histological scores referred to small intestine of 12 weeks old PTPMT1 Wt (N=4) and PTPMT1 ΔT (N=4) mice. (**d**) Frequencies of TBET-expressing CD4^+^ cells in small intestine lamina propria (siLP, PTPMT1 Wt N=4, PTPMT1 ΔT N=3), mesenteric lymph nodes (mLN, PTPMT1 Wt N=4, PTPMT1 ΔT N=4) and spleen (PTPMT1 Wt N=4, PTPMT1 ΔT N=4). (**e**) Schematic of the *Listeria monocytogenes* (Lm) infection model in PTPMT1 Wt and PTPMT1 ΔT mice. (**f**) Frequencies of CD4^+^ cells 7 days in circulation post infection with Lm from PTPMT1 Wt (N=4) and PTPMT1 ΔT (N=5) mice. (**g**) Frequencies of CD4^+^CD62L^−^CD44^+^ T cells in circulation 7 days post infection with Lm from PTPMT1 Wt (N=4) and PTPMT1 ΔT (N=5) mice. (**h**) Frequencies of CD4^+^ cells expressing TBET (left panel, PTPMT1 Wt N=4 and PTPMT1 ΔT N=4) and IFN γ (right panel, PTPMT1 Wt N=4 and PTPMT1 ΔT N=4) after 4 days inTh1 differentiating culture conditions. (**i**) Frequencies of RORγt^+^ expressing CD4^+^ cells in small intestine lamina propria (siLP, PTPMT1 Wt N=4, PTPMT1 ΔT N=3), mesenteric lymph nodes (mLN, PTPMT1 Wt N=4, PTPMT1 ΔT N=4) and spleen (PTPMT1 Wt N=4, PTPMT1 ΔT N=4). (**j**) Schematic of the EAE Model (left panel). Mice were immunized with 200 μg of myelin oligodendrocyte glycoprotein peptide emulsified in complete Freund’s adjuvant by intraperitoneal injections together with 200 ng pertussis toxin (PTX) at the time of immunization and 24 h later injected subcutaneously. Disease severity scored daily from day 7 onwards (6 mice per group) is shown (right panel). (**k**) Frequencies (left panel, PTPMT1 Wt N=4 and PTPMT1 ΔT N=5 mice) and numbers (right panel, PTPMT1 Wt N=4 and PTPMT1 ΔT N=5 mice) of brain-infiltrating TCRβ^+^ CD4^+^ T cells detected by flow cytometry 15 days after EAE immunization. (**l**) Frequencies of CD4^+^ cells expressing RORγt (left panel, PTPMT1 Wt N=4 and PTPMT1 ΔT N=4) and IL-17 (right panel, PTPMT1 Wt N=4 and PTPMT1 ΔT N=4) after 4 days in Th17 differentiating culture conditions. (**m**) Frequencies of CD4^+^ cells expressing TBET (PTPMT1 Wt N=4 and PTPMT1 ΔT N=4), IFNγ (PTPMT1 Wt N=4 and PTPMT1 ΔT N=4), RORγt (PTPMT1 Wt N=4 and PTPMT1 ΔT N=4) and IL-17A (PTPMT1 Wt N=4 and PTPMT1 ΔT N=4) after 4 days in Th1, Th2, Th17 and Treg differentiating culture conditions. (**n**) Representative overlays showing PTPMT1 Wt and PTPMT1 ΔT CD4^+^ cells proliferation after 4 days in Th1, Th2, Th17 and Treg differentiating culture conditions. (**o**) Frequencies of CD8^+^, CD4^+^, CD4^+^ FOXP3^+^, CD4^+^ Tbet^+^ in kidney (PTPMT1 Wt N=3 and PTPMT1 ΔT N=3), liver (PTPMT1 Wt N=3 and PTPMT1 ΔT N=3), lung (PTPMT1 Wt N=3 and PTPMT1 ΔT N=3) and brain (PTPMT1 Wt N=3 and PTPMT1 ΔT N=3). (**p**) Frequencies of inflammatory monocytes, classic macrophages, neutrophils and dendritic cells in kidney (PTPMT1 Wt N=3 and PTPMT1 ΔT N=3), liver (PTPMT1 Wt N=3 and PTPMT1 ΔT N=3), lung (PTPMT1 Wt N=3 and PTPMT1 ΔT N=3) and brain (PTPMT1 Wt N=3 and PTPMT1 ΔT N=3). Data shown as mean ± SEM of three independent experiments unless indicated. Statistical comparisons between two groups were calculated using an unpaired two-tailed Student’s t test. Log-rank (Mantel-Cox) test was performed to compare survival curves in panel (**b**). Exact p-values are indicated. Source data

PTPMT1 ΔT mice (*Ptpmt1*^fl/fl^*CD4*-Cre^*tg*^*/*^*+*^*)*, which lack the expression of *Ptpmt1* in all T cells, showed progressively lower weight gain and earlier weight loss during ageing compared to PTPMT1 wild type (WT; *Ptpmt1*^fl/fl^*CD4-*Cre^+/+^) littermates (Fig. 1a). This phenotype was associated with reduced median lifespan, which was on average 7 months shorter (PTPMT1 WT 128 weeks versus PTPMT1 ΔT 90 weeks; Fig. 1b). Reduced body weight and lifespan was consistent in males and females, although females showed a milder and a later-onset phenotype (Extended Data Fig. 1a,b). Autoptic examinations of PTPMT1 ΔT mice reported anal prolapse and intestinal inflammation as the most likely cause of death. To investigate this finding further, we analysed the small intestines of PTPMT1 WT and PTPMT1 ΔT mice by histopathology. H&E, periodic acid–Schiff (PAS) staining and anti-CD45 staining revealed altered crypt structures and reduced goblet cell numbers associated with infiltration of CD45^+^ immune cells in vast areas of the small intestine lamina propria (siLP) of PTPMT1 ΔT mice (Fig. 1c,d and Extended Data Fig. 1c). To define the cellular composition and transcriptional profile of the immune infiltrate in PTPMT1 ΔT mice, CD45^+^ cells sorted from the siLP of PTPMT1 WT and PTPMT1 ΔT mice were analysed by single-cell RNA sequencing (scRNA-seq). We identified 22 clusters covering the main immune cell subsets (Fig. 1e and Supplementary Fig. 1a). A comparison of cluster proportions showed that PTPMT1 ΔT samples had increased frequencies of macrophages, dendritic cells and neutrophils (Fig. 1e). These subsets showed an elevated inflammatory profile as revealed by interferon-γ (IFNγ) and tumour necrosis factor (TNF) signalling scores (Fig. 1f and Supplementary Fig. 1b). Accumulation of these pro-inflammatory myeloid cell subsets in siLP, mesenteric lymph nodes (mLNs) and spleen was also confirmed by cytofluorometry analysis (Fig. 1g). scRNA-seq analysis revealed instead a reduction in the infiltration of the immunosuppressive T_reg_ cell subset (Fig. 1e). Overall, these results show that the loss of cardiolipin leads to inflammation in the gut with a significant increase in inflammatory myeloid cells.

### Helper T cell imbalance and T_reg_ impairment drive gut inflammation in PTPMT1 ΔT mice

We then investigated the effects of *Ptpmt1* deletion in helper T cell subsets to determine the cause of the inflammatory phenotype of the PTPMT1 ΔT mice. T_reg_ cells, which exclusively express the transcription factor FOXP3, are crucial to control gut homeostasis thanks to their immunosuppressive capacity^17,18^. Thus, we investigated whether an impaired phenotype of the immunosuppressive population of T_reg_ cells could mediate the inflammatory response observed in the PTPMT1 ΔT mice. The frequency of FOXP3^+^ CD4^+^ cells in the siLP, mLNs and spleen of PTPMT1 ΔT mice was lower compared to WT littermates, alongside reduced expression of T_reg_ markers CD357 (GITR) and CD304 (neuropilin-1 or NRP1) in the splenic T_reg_ cells (Fig. 1h,i). These data further corroborated the lower T_reg_ functional score shown by the scRNA-seq analysis (Fig. 1j and Supplementary Fig. 1c). Moreover, in vitro culture of PTPMT1 ΔT CD4^+^ T cells under T_reg_ cell differentiating conditions showed reduced expression of FOXP3, CD25, CD152 (CTLA-4) and CD278 (ICOS) compared to PTPMT1 WT cells, revealing that cardiolipin deficiency triggers T_reg_ dysfunction in a cell-autonomous manner (Fig. 1k). Finally, in vivo transfer of WT T_reg_ cells was sufficient to extend lifespan and rescue the severe phenotype of PTPMT1 ΔT mice (Fig. 1l). The results of this T_reg_ rescue experiment further sustain the specific role of T_reg_ cells in establishing (or failing to establish) the tolerogenic environment in the gut of PTPMT1 ΔT mice.

Previous reports suggested that mitochondrial deficiencies also cause inflammation by shifting the phenotype of helper T cell subsets towards a type 1 helper T (T_H_1) cell phenotype^50^. The cytofluorometry immunophenotyping revealed similar frequencies of CD4^+^ cells expressing T-box protein expressed in T cells (TBET, a transcription factor specific for T_H_1 cells) in the siLP, mLNs or spleen of PTPMT1 ΔT mice compared to WT littermates (Extended Data Fig. 1d). Moreover, when mice were challenged intravenously with an *L. monocytogenes* infection that drives a T_H_1-mediated response, PTPMT1 ΔT CD4^+^ cells failed to proliferate or differentiate into effector CD4^+^CD44^+^CD62L^−^ cells (Extended Data Fig. 1e–g). TBET expression was unaltered, while IFNγ expression was even reduced in an in vitro model where CD4^+^ cells were activated in T_H_1 differentiating conditions (Extended Data Fig. 1h). In addition to T_H_1, the T_H_17 subset of helper T cells is often implicated in the development of colitis^51^. The immunophenotyping showed no differences in the frequencies of cells expressing retinoic acid-related orphan receptor γt (RORγt, a T_H_17-specific transcription factor; Extended Data Fig. 1i). To test in vivo the activation and function of T_H_17 cells, we induced experimental autoimmune encephalomyelitis (EAE), the severity of which depends mainly on T_H_17 cells^52^, in PTPMT1 WT and PTPMT1 ΔT mice (Extended Data Fig. 1j). Notably, PTPMT1 ΔT mice developed only a mild phenotype with a low pathology score and minimal infiltration of pathogenic CD4^+^ T cells in the brain compared to PTPMT1 WT littermates (Extended Data Fig. 1j,k). Moreover, when cultured in vitro towards a T_H_17 phenotype, PTPMT1 ΔT cells (while expressing RORγt similarly to WT cells) failed to produce the inflammatory cytokine IL-17a (Extended Data Fig. 1l). Overall, our analysis revealed that, while PTPMT1 ΔT cells can differentiate in T_H_1 and T_H_17 cells, they cannot produce the pro-inflammatory cytokines IFNγ and IL-17a. Moreover, no transdifferentiation towards T_H_1 or T_H_17 was observed in other helper T cell subsets, all showing a proliferative defect (Extended Data Fig. 1m,n).

Finally, although lymphopenia in PTPMT1 ΔT mice extended beyond the small intestines, affecting CD8^+^, CD4^+^, FOXP3^+^ and TBET^+^ cells across multiple organs, including the liver, kidney, lung and brain, the inflammatory phenotype was only present in the intestines (Extended Data Fig. 1o,p). This finding suggests that the unique microenvironment of the gut is critical for the development of inflammation in PTPMT1 ΔT mice. Taken together, our analyses strongly suggest that the loss of cardiolipin causes gut inflammation by impairing T_reg_ cell function rather than through the overactivation of T_H_1 or T_H_17 cells.

### PTPMT1 is necessary for T_reg_ immunosuppressive function

**Fig. 2:**
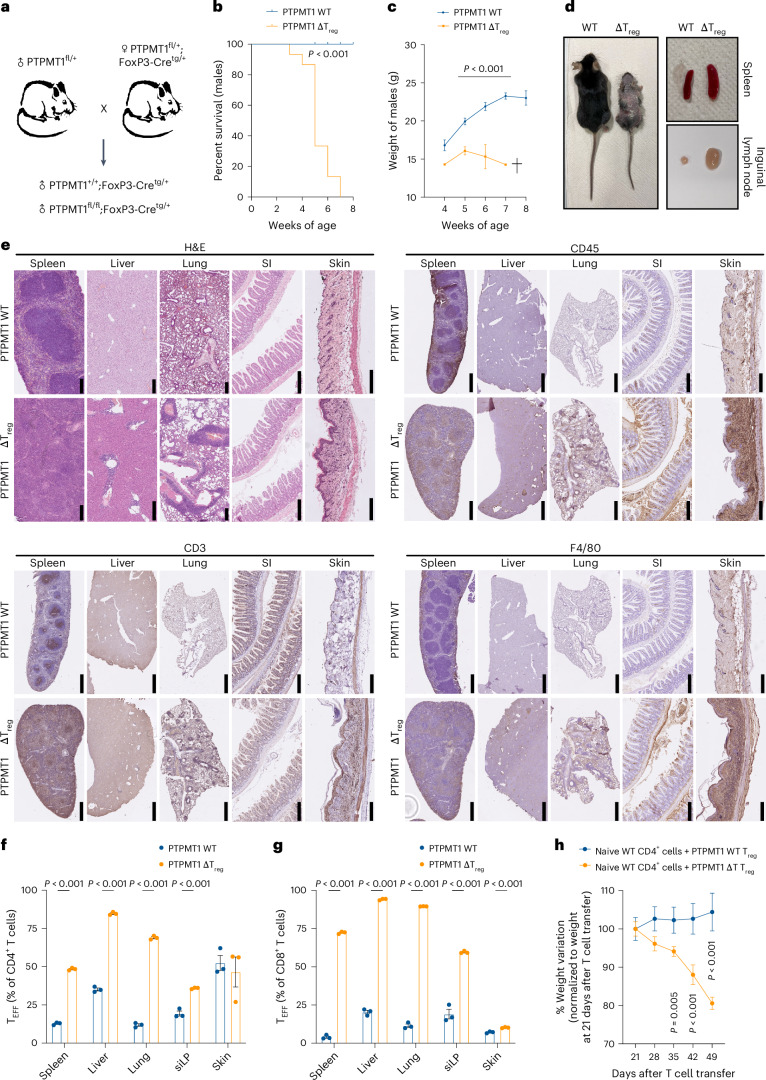
PTPMT1 is necessary for T_reg_ cell immunosuppression. **a**, Schematic depicting the generation of PTPMT1^fl/fl^: FoxP3-Cre^tg/+^ (PTPMT1 ΔT_reg_) mouse strain. **b**, Kaplan–Meier survival curve of PTPMT1 WT (*N* = 18) and PTPMT1 ΔT_reg_ (*N* = 15) male mice. **c**, Body weight curve of PTPMT1 WT (*N* = 18) and PTPMT1 ΔT_reg_ (*N* = 7) male mice. **d**, Representative pictures of PTPMT1 WT and PTPMT1 ΔT_reg_ mice euthanized at 6 weeks (left) and their spleens and inguinal lymph nodes (right). **e**, Representative microscopy images of spleen, liver, lung, small intestine (SI) and skin sections stained with H&E and immunostained with antibodies against CD45, CD3 and F4/80. Scale bars, 250μm. **f**, Frequencies of CD44^+^CD62L^−^CD4^+^ T effector cells in the spleen, liver, lung, siLP and skin from PTPMT1 WT and PTPMT1 ΔT_reg_ mice. **g**, Frequencies of CD44^+^CD62L^−^CD8^+^ T effector cells in the spleen, liver, lung, siLP and skin from PTPMT1 WT and PTPMT1 ΔT_reg_ mice. **h**, Body weight curve of mice injected with naive WT CD4^+^ cells plus in vitro generated PTPMT1 WT T_reg_ (*N* = 3) or naive WT CD4^+^ cells plus in vitro generated PTPMT1 ΔT_reg_ (*N* = 3). Data are shown as the mean ± s.e.m. of three independent experiments unless indicated. Statistical comparisons between two groups were calculated by using an unpaired two-tailed Student’s *t*-test. Log-rank (Mantel–Cox) test was performed to compare survival curves in **b**. Exact *P* values are indicated. Source data

**Extended Data Fig. 2:**
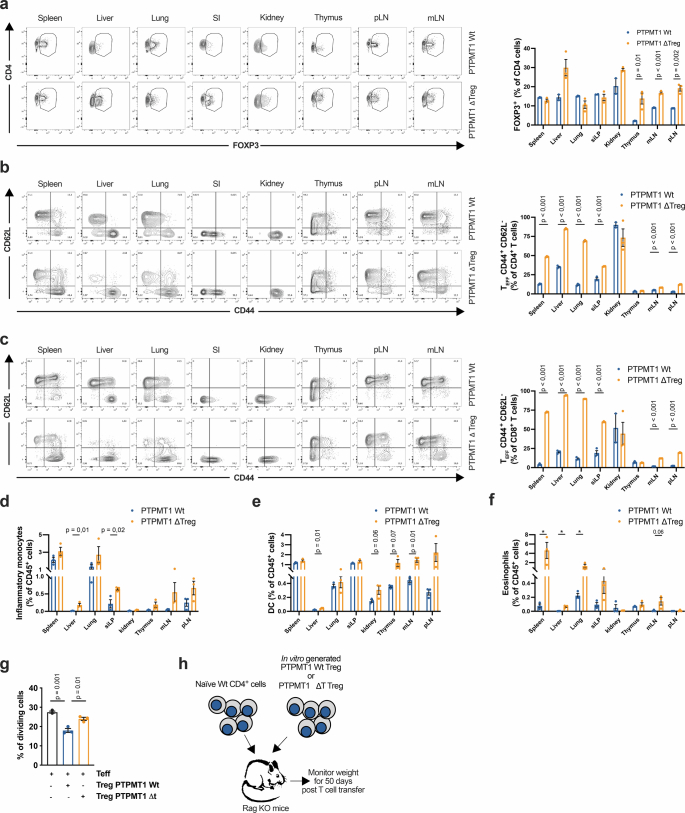
PTPMT1 loss in Treg cells induces multi-organ autoimmunity. (**a**) Representative flow cytometry dot plots (left) and frequencies (right) of CD4^+^ FOXP3^+^ T cells in the indicated tissues of PTPMT1 Wt and PTPMT1 ΔTreg mice. (**b**) Representative flow cytometry dot plots (left) and frequencies (right) of CD62L^−^CD44^+^CD4^+^ T cells in the indicated tissues of PTPMT1 Wt and PTPMT1 ΔTreg mice (**c**) Representative flow cytometry dot plots (left) and frequencies (right) of CD62L^−^CD44^+^CD8^+^ T cells in the indicated tissues of PTPMT1 Wt and PTPMT1 ΔTreg mice. (**d**) Frequencies of infiltrating monocytes in the indicated tissues of PTPMT1 Wt and PTPMT1 ΔTreg mice. (**e**) Frequencies of dendritic cells (DC) in the indicated tissues of PTPMT1 Wt and PTPMT1 ΔTreg mice. (**f**) Frequencies of eosinophils in the indicated tissues of PTPMT1 Wt and PTPMT1 ΔTreg mice. (**g**) Frequencies of CD8^+^ T cells reaching division 4 in *in vitro* suppression assay. (**h**) Schematic of *in vivo* T cell-mediated colitis model. Data shown as mean ± SEM of three independent experiments unless indicated. Statistical comparisons between two groups were calculated using an unpaired two-tailed Student’s t test. Exact p-values are indicated. Source data

To test the specific requirement for PTPMT1 in T_reg_ cells, we generated a new mouse model where *Ptpmt1*^fl/fl^ mice were crossed with *Foxp3-Yfp*-Cre mice to achieve *Ptpmt1* deletion exclusively in T_reg_ cells (PTPMT1 WT T_reg_ versus PTPMT1 ΔT_reg_; Fig. 2a). Male PTPMT1 ΔT_reg_ mice developed a severe phenotype, manifested by short lifespan (maximum 7 weeks; Fig. 2b), reduced body weight (Fig. 2c) and small overall size. In addition, they developed scarred skin, splenomegaly and lymphadenitis, consistent with autoimmunity (Fig. 2d). Of note, only male mice developed the phenotype as the strain expresses the Cre recombinase under the control of the natural *Foxp3* promoter, which is located on the X chromosome. Due to the presence of the *Foxp3*-Cre allele always in one copy and due to random X-chromosome inactivation, half of the T_reg_ cells in females do not express Cre and maintain a WT phenotype. To investigate further the autoimmune phenotype in the PTPMT1 ΔT_reg_ mice, the spleen, liver, lung, small intestines and skin were analysed by histology and immunohistochemistry (Fig. 2e). H&E staining revealed perturbed tissue architecture (Fig. 2e) with immune infiltrate (Fig. 2e; CD45) including T cells (Fig. 2e; CD3) and macrophages (Fig. 2e; F4/80) across all organs examined. Although FOXP3^+^ cells were still present at a similar frequency in PTPMT1 ΔT_reg_ mice compared to control animals (Extended Data Fig. 2a), their function was compromised as all remaining CD8^+^ and CD4^+^ T cells acquired a hyperactivated effector phenotype (CD44^+^CD62L^−^; Fig. 2f,g and Extended Data Fig. 2b,c). Infiltration of inflammatory monocytes, dendritic cells and eosinophils was also prominent (Extended Data Fig. 2d–f). To test T_reg_ immunosuppressive function, we performed an in vitro suppression assay. We found that T_reg_ cells lacking PTPMT1 could not block the proliferation of CD8^+^ effector cells as efficiently as WT T_reg_ cells (Extended Data Fig. 2g). Moreover, in an in vivo colitis model, naive CD4^+^ T cells were injected simultaneously with either PTPMT1 WT T_reg_ or PTPMT1 ΔT_reg_ cells into recombination activating gene 2 (RAG2) knockout (KO) mice (Extended Data Fig. 2h). Only mice co-injected with PTPMT1 ΔT_reg_ lost weight due to colitis, further confirming that the loss of PTPMT1 impacts the immunosuppressive function of T_reg_ cells (Fig. 2h). Collectively, our analysis shows that PTPMT1 is essential for T_reg_ cells to limit excessive inflammation and autoimmunity and maintain tissue homeostasis.

### The microbiota modulates the severity of gut inflammation in PTPMT1 ΔT mice

**Fig. 3:**
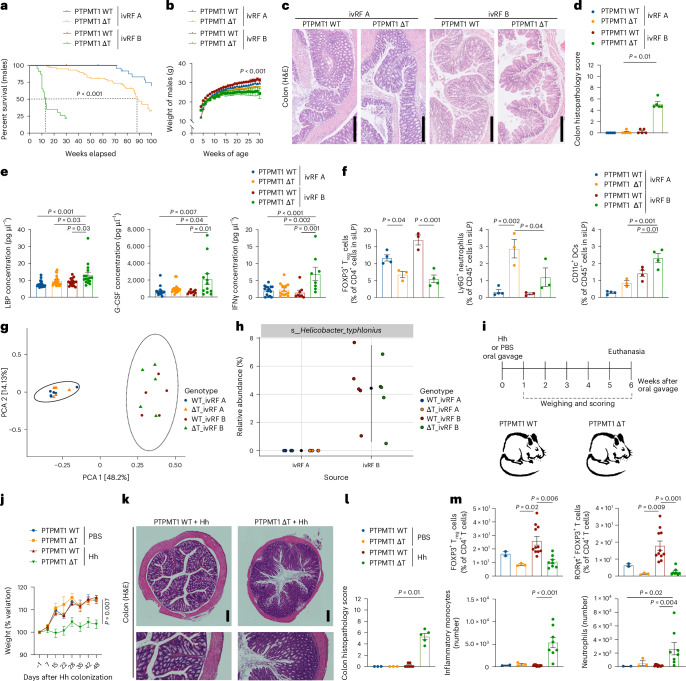
PTPMT1 ΔT sensitizes mice to pathobiont colonization. **a**, Kaplan–Meier survival curve of PTPMT1 WT (ivRF A, *N* = 30; ivRF B, *N* = 16) and PTPMT1 ΔT (ivRF A, *N* = 28; ivRF B, *N* = 17) male mice. **b**, Body weight curve of PTPMT1 WT (ivRF A, *N* = 30; ivRF B, *N* = 16) and PTPMT1 ΔT (ivRF A, *N* = 28; ivRF B, *N* = 17) male mice. **c**, Representative images of colon sections from PTPMT1 WT and PTPMT1 ΔT mice stained with H&E from ivRF A and ivRF B. Scale bars, 250 μm. **d**, Histopathology score of colon from PTPMT1 WT and PTPMT1 ΔT mice from ivRF A and ivRF B (*N* = 5 mice per genotype). **e**, Serum concentration levels of lipopolysaccharide binding protein (LBP; PTPMT1 WT ivRF A, *N* = 22; ivRF B, *N* = 17 and PTPMT1 ΔT ivRF A, *N* = 20; ivRF B, *N* = 16), G-CSF (PTPMT1 WT ivRF A, *N* = 17; ivRF B, *N* = 9 and PTPMT1 ΔT ivRF A, *N* = 17; ivRF B, N = 12) and IFNγ (PTPMT1 WT ivRF A, *N* = 15; ivRF B, *N* = 8 and PTPMT1 ΔT ivRF A, *N* = 14; ivRF B, *N* = 8). **f**, Expression of FOXP3^+^ T_reg_ cells (PTPMT1 WT ivRF A, *N* = 4; ivRF B, *N* = 3 and PTPMT1 ΔT ivRF A, *N* = 3; ivRF B, *N* = 4), Ly6G^+^ neutrophils (PTPMT1 WT ivRF A, *N* = 4; ivRF B, *N* = 3 and PTPMT1 ΔT ivRF A, *N* = 3; ivRF B, *N* = 3) and CD11c^+^ dendritic cells (PTPMT1 WT ivRF A, *N* = 4; ivRF B, *N* = 3 and PTPMT1 ΔT ivRF A, *N* = 4; ivRF B, *N* = 4) in the siLP. **g**, Principal component analysis (PCA) plot of the gut microbiota composition in stool samples collected from PTPMT1 WT (ivRF A, *N* = 5; ivRF B, *N* = 5) and PTPMT1 ΔT (ivRF A, *N* = 5; ivRF B, *N* = 5) mice. **h**, Relative abundance of *H. typhlonius* in stool samples collected from PTPMT1 WT (ivRF A, *N* = 5; ivRF B, *N* = 5) and PTPMT1 ΔT (ivRF A, *N* = 5; ivRF B, *N* = 5) mice. **i**, Schematic of the *H. hepaticus* (Hh) infection model in PTPMT1 WT and PTPMT1 ΔT mice. **j**, Percentage body weight variation of PTPMT1 WT + PBS (*N* = 3), PTPMT1 ΔT + PBS (*N* = 3), PTPMT1 WT + Hh (*N* = 11) and PTPMT1 ΔT + Hh (*N* = 8) male mice compared to weight one day before colonization. **k**, Representative H&E images of colon sections from PTPMT1 WT and PTPMT1 ΔT mice gavaged with Hh. Scale bars, 250 μm. **l**, Colon histopathology scores from PTPMT1 WT and PTPMT1 ΔT mice gavaged with PBS or Hh (PTPMT1 WT: PBS, *N* = 3; Hh, *N* = 7 and PTPMT1 ΔT: PBS, *N* = 3; Hh, *N* = 5). **m**, Frequencies of FOXP3^+^ T_reg_, FOXP3^+^ RORγt T_reg_ and numbers of inflammatory monocytes and neutrophils in the colon of PTPMT1 WT and PTPMT1 ΔT mice gavaged with PBS or Hh (PTPMT1 WT: PBS, *N* = 2; Hh, *N* = 11 and PTPMT1 ΔT: PBS, *N* = 3; Hh, *N* = 8). Data are shown as the mean ± s.e.m. of three independent experiments unless indicated. Statistical comparisons between two groups were calculated by using an unpaired two-tailed Student’s *t*-test. Statistical comparison among more than two groups were calculated using one-way analysis of variance (ANOVA) Kruskal–Wallis test with Dunn’s or Tukey’s correction. Exact *P* values are indicated. Source data

**Extended Data Fig. 3:**
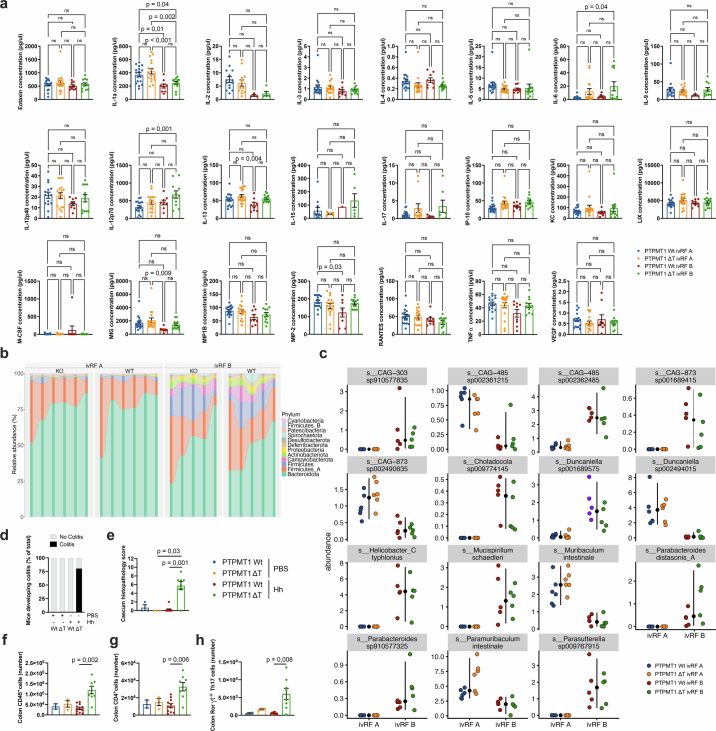
Microbiota modulates the severity of gut inflammation in PTPMT1 ΔT mice. (**a**) Serum concentration levels of the indicated cytokines in PTPMT1 Wt (ivRF A, N=17; ivRF B, N=9) and PTPMT1 ΔT (ivRF A, N=16; ivRF B, N=12). (**b**) Relative abundance of gut bacteria phyla in stool samples collected from PTPMT1 Wt and PTPMT1 ΔT mice from ivRF A and ivRF B. (**c**) Relative abundance of different gut microbiota species in stool samples collected from PTPMT1 Wt and PTPMT1 ΔT mice from ivRF A and ivRF B. (**d**) Percentage of mice developing colitis 28 days post PBS or *H.hepaticus* gavage in PTPMT1 Wt and PTPMT1 ΔT mice. (**e**) Caecum histopathology scores from PTPMT1 Wt and PTPMT1 ΔT mice gavaged with PBS or *H.hepaticus* (PTPMT1 Wt: PBS N=3, Hh N=11; PTPMT1 ΔT; PBS, N=3, Hh N=8). (**f**) Numbers of CD45^+^ cells in the colon of PTPMT1 Wt and PTPMT1 ΔT mice gavaged with PBS or *H.hepaticus* (PTPMT1 Wt: PBS N=2, Hh N=11; PTPMT1 ΔT; PBS, N=3, Hh N=8). (**g**) Numbers of CD4^+^ cells in the colon of PTPMT1 Wt and PTPMT1 ΔT mice gavaged with PBS or *H.hepaticus* (PTPMT1 Wt: PBS N=2, Hh N=11; PTPMT1 ΔT; PBS, N=3, Hh N=8). (**h**) Numbers of RORγt^+^ Th17 cells in the colon of PTPMT1 Wt and PTPMT1 ΔT mice gavaged with PBS or *H.hepaticus* (PTPMT1 Wt: PBS N=2, Hh N=11; PTPMT1 ΔT; PBS, N=3, Hh N=8). Data shown as mean ± SEM of three independent experiments unless indicated. Statistical comparison among more than two groups were calculated using the Kruskall-Wallis test with Dunn’s correction. Exact p-values are indicated. Source data

Despite widespread T_reg_ deficiency, PTPMT1 ΔT mice showed the most severe phenotypes in the gut (Fig. 1 and Extended Data Fig. 1o,p). Here, T_reg_ cells are critical to establish a tolerogenic environment in response to the intestinal microbial challenge^3^. We hypothesized that the overall microbiota composition or the presence/absence of specific microbial species could exacerbate the gut inflammatory phenotype of PTPMT1 ΔT mice. Thus, to test this hypothesis, we re-derived the PTPMT1 ΔT mouse colony via in vitro fertilization and housed it in a completely independent in vivo research facility (ivRF B) where mice, through their foster mothers, established a different microbiota. Strikingly, the PTPMT1 ΔT mice housed in ivRF B displayed earlier onset of severe symptoms, including anal prolapses and death starting from 2 months of age, compared to the mice in ivRF A where lifespan reduction appeared much later (Fig. 3a). Mouse weight differences (Fig. 3b), histopathology of mid colon (Fig. 3c,d), gut permeability (measured by lipopolysaccharide binding protein (LBP) concentration in the serum) as well as systemic inflammatory profile (G-CSF, IFNγ and other cytokine serum concentrations) were exacerbated in the PTPMT1 ΔT mice housed in ivRF B (Fig. 3e and Extended Data Fig. 3a). The phenotype was observed in both the husbandry locations with similar frequencies of lamina propria-resident FOXP3^+^ T_reg_ cells and neutrophils but higher infiltration of dendritic cells in PTPMT1 ΔT mice in ivRF B (all analysed when mice were 10–12 weeks old; Fig. 3f). Despite a similar genetic and metabolic profile of PTPMT1 ΔT mice in the two facilities, the more severely inflamed tissue pathology in ivRF B prompted us to investigate gut microbiota composition as an environmental factor influencing the different outcomes. Metagenomics analysis of microbiota from stool collected from 12-week-old PTPMT1 WT and PTPMT1 ΔT mice from both ivRFs showed no dysbiosis between PTPMT1 WT and PTPMT1 ΔT mice ruling out genotype-specific microbiota alterations (Fig. 3g and Extended Data Fig. 3b). Nevertheless, this analysis showed that, irrespective of the genotype, mice from the two ivRFs hosted different microbiota (Fig. 3g). Further analysis showed that the differences between the two ivRFs could be traced back to only 15 different micro-organisms (Extended Data Fig. 3c). Among them, *H. typhlonius* in ivRF B caught our attention (Fig. 3h). *H. typhlonius* is a classic pathobiont able to induce severe colitis and inflammation only in immunocompromised and IL-10-deficient mice^14,15^. Altogether, our data identified specific microbial species potentially influencing the intensity of gut inflammation in PTPMT1 ΔT mice.

### PTPMT1 ΔT mice are susceptible to pathobiont colonization

We therefore set out to investigate whether re-inoculation of *Helicobacter* could recapitulate the severe phenotype observed in ivRF B. Twelve-week-old PTPMT1 WT and PTPMT1 ΔT mice from ivRF A (devoid of *Helicobacter* species) were gavaged with either PBS or *H. hepaticus*, a close relative strain to *H. typhlonius* from the *Helicobacter* species, widely used for mouse colonization studies^12,16^. Mouse weight and phenotypic manifestation of colitis were recorded weekly after colonization (Fig. 3i). Unlike PBS-gavaged PTPMT1 WT, PTPMT1 ΔT mice and *H. hepaticus*-gavaged PTPMT1 WT mice, *H. hepaticus*-gavaged PTPMT1 ΔT failed to gain weight (Fig. 3j) and developed colitis (Extended Data Fig. 3d). Six weeks after colonization, histopathological analysis of colon and caecum from *H. hepaticus*-gavaged PTPMT1 ΔT mice showed clinical signs of colitis with higher histopathology score driven by immune infiltration, crypt alterations and goblet cell depletion (Fig. 3k,l and Extended Data Fig. 3e). Cytofluorometric analysis confirmed CD45^+^ cell infiltration in *H. hepaticus*-gavaged PTPMT1 ΔT colon lamina propria with higher CD4^+^ T cell infiltrate (Extended Data Fig. 3f,g). Of note, the analysis of CD4^+^ T cell subsets showed that in colons from *H. hepaticus*-gavaged PTPMT1 WT mice, there was an increase in the frequencies of FOXP3^+^ and RORγt^+^ FOXP3^+^ CD4^+^ T_reg_ cells that are critical for controlling *H. hepaticus* pathology (Fig. 3m). Conversely, in colon from *H. hepaticus*-gavaged PTPMT1 ΔT mice, there was no increase in T_reg_ cell frequency. Instead, we observed an accumulation of pathogenic RORγt^+^ T_H_17 cells (Extended Data Fig. 3h) together with neutrophils and inflammatory monocytes (Fig. 3m). Overall, we show that PTPTM1 deficiency in T cells sensitizes mice to pathobiont colonization and dictates severity of colitis clinical outcome in a T_reg_ cell-dependent manner.

### Cardiolipin deficiency but not PGP accumulation drives the phenotype of PTPMT1 ΔT cells

**Fig. 4:**
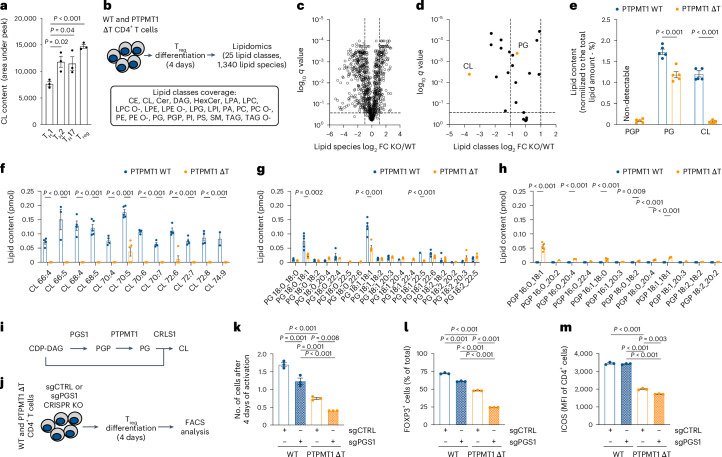
Cardiolipin deficiency and not PGP accumulation drives the phenotype of PTPMT1 ΔT cells. **a**, Cardiolipin (CL) content in WT CD4^+^ T cells differentiated in vitro in T_H_1, T_H_2, T_H_17 and T_reg_ cells (*N* = 3 per group). **b**, Schematics of lipidomics analysis of PTPMT1 WT and PTPMT1 ΔT T_reg_ cells covering 25 lipid classes and 1,340 lipid species. **c**, log_2_fold change (FC) PTPMT1 ΔT/ PTPMT1 WT of lipid species in cells activated and differentiated as in **b**. **d**, log_2_FC PTPMT1 ΔT/PTPMT1 WT of lipid classes in cells activated and differentiated as in **b**. **e**, PGP, PG and CL lipid content in PTPMT1 WT and PTPMT1 ΔT T_reg_ cells (*N* = 5 per genotype). **f**, Quantification of CL lipid species in PTPMT1 WT and PTPMT1 ΔT T_reg_ cells (*N* = 5 per genotype). **g**, Quantification of PG lipid species containing stearic acid, oleic acid and linoleic acid in PTPMT1 WT and PTPMT1 ΔT T_reg_ cells (*N* = 5 per genotype). **h**, Quantification of PG lipid species in PTPMT1 WT and PTPMT1 ΔT T_reg_ cells (*N* = 5 per genotype). **i**, Depiction of CL synthesis pathway including lipid species and proteins involved. **j**, Schematic of experimental design to generate sgCTRL or sgPGS1 CRISPR KO in naive PTPMT1 WT and PTPMT1 ΔT CD4^+^ T cells before T_reg_ cell differentiation. **k**, Number of cells of the indicated genotypes after 4 days of T_reg_ cell differentiation. **l**, FOXP3 expression (%) in cells of the indicated genotypes after 4 days of T_reg_ cell differentiation. **m**, ICOS expression (MFI) in cells of the indicated genotypes after 4 days of T_reg_ cell differentiation. Data are shown as the mean ± s.e.m. of three independent experiments unless indicated. Statistical comparisons between two groups were calculated by using an unpaired two-tailed Student’s *t*-test. Statistical comparison was calculated using one-way ANOVA in **c** and **d**, with Tukey’s correction in **k**–**m**. Exact *P* values are indicated. Source data

**Extended Data Fig. 4:**
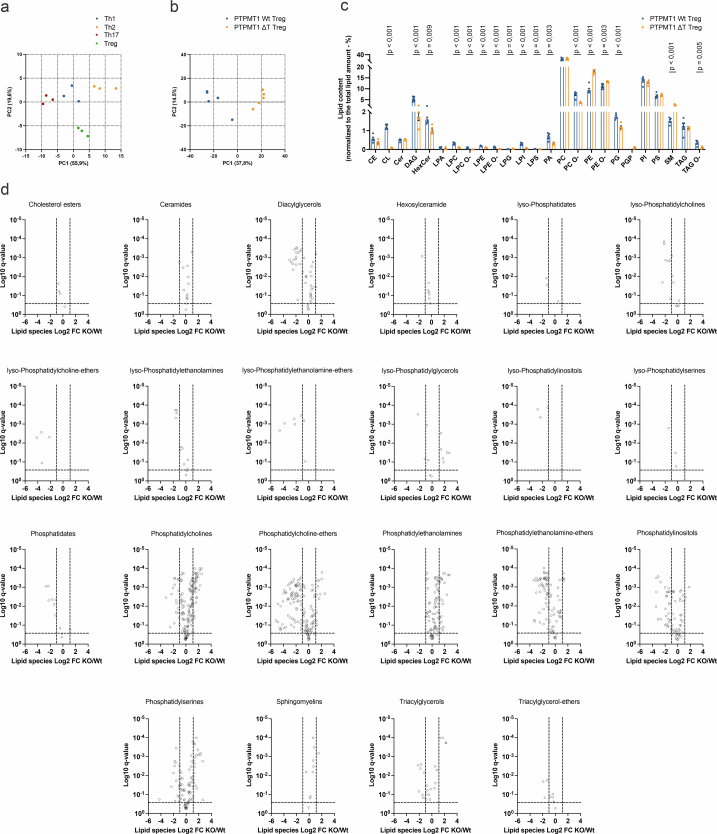
PTPMT1 deficiency triggers a broad rewiring of the cellular lipidome. (**a**) Principal component analysis of lipidomic profile of CD4^+^ Helper T cell subsets. (**b**) Principal component analysis of lipidomic profile of PTPMT1 Wt (N=5) and PTPMT1 ΔT (N=5) Treg cells. (**c**) Lipid content profile (lipid classes) of PTPMT1 Wt (N=5) and PTPMT1 ΔT (N=5) Treg cells. (**d**) Volcano plots depicting Log2FC PTPMT1 ΔT/ PTPMT1 Wt of lipid species split lipid classes. Data shown as mean ± SEM. Statistical comparisons between two groups were calculated using an unpaired two-tailed Student’s t test. Statistical comparison was calculated using One-Way ANOVA in panel (**d**). Exact p-values are indicated. Source data

WT T_reg_ cells are characterized by a unique lipidomic profile with the enrichment of cardiolipin compared to the other helper T cell subsets (Fig. 4a and Extended Data Fig. 4a), which makes T_reg_ cells particularly sensitive to disturbances of the cardiolipin synthesis pathway. PTPMT1 mediates the conversion of phosphatidylglycerophosphate (PGP) to phosphatidylglycerol (PG), the limiting step reaction for cardiolipin synthesis. Its ablation leads to cardiolipin deficiency but can lead to the accumulation of its substrate PGP and changes to the mitochondrial lipidome. Whether the effects of PTPMT1 deficiency depend on cardiolipin deficiency or on the accumulation of PGP is unclear. To determine the effects of PTPMT1 loss in T_reg_ cell lipidome, we performed an untargeted lipidomic analysis of WT and PTPMT1 ΔT_reg_ cells. Our dataset included 25 lipid classes and 1,340 lipid species (Fig. 4b). PTPMT1 deficiency induced a broad rewiring of the cellular lipidome (Fig. 4c and Extended Data Fig. 4b–d). Focusing on lipid classes involved in cardiolipin biosynthesis, cardiolipins were the most strongly downregulated species in PTPMT1 ΔT_reg_ cells (Fig. 4d,e). Consistent with the enzymatic function of PTPMT1, its deletion also reduced PGs (Fig. 4e,g). PGP species were undetectable in WT cells, consistent with their role as short-lived intermediates rapidly converted into PG by PTPMT1 (Fig. 4e,h). In PTPMT1 ΔT cells, PGP levels increased but only modestly (Fig. 4e,h). This limited, nonlinear accumulation of PGP relative to the reduction in PG suggests that PGP may be chemically unstable and either degraded or diverted into alternative lipid species.

To evaluate whether this modest PGP accumulation could be toxic, we deleted phosphatidylglycerophosphate synthase 1 (PGS1) using CRISPR–Cas9 in both PTPMT1 WT and PTPMT1 ΔT_reg_ cells. PGS1 converts cytidine diphosphate diacylglycerol (CDP-DAG) into PGP; its deletion in WT cells reduces PGP and thus diminishes cardiolipin synthesis downstream. In PTPMT1 ΔT_reg_ cells, PGS1 KO eliminates PGP accumulation in the context of pre-existing cardiolipin deficiency (Fig. 4i). Therefore, we generated WT, PGS1 KO, PTPMT1 KO and PGS1–PTPMT1 double-knockout (DKO) T cells, which we then activated and differentiated into T_reg_ cells (Fig. 4j). PGS1 KO alone impaired proliferation and reduced FOXP3^+^ CD4^+^ T_reg_ frequencies, consistent with the phenotype of cardiolipin-deficient T_reg_ cells (Fig. 4k–m). Notably, PGS1–PTPMT1 DKO cells displayed an even more severe phenotype, with further reduced proliferation and lower expression of FOXP3 and other critical proteins for T_reg_ cell function including ICOS compared to PTPMT1 ΔT_reg_ cells (Fig. 4k–m).

Together, these results indicate that PGP is not toxic. Instead, we speculate that PGP may retain a partial compensatory function in membrane organization under conditions of cardiolipin deficiency.

### Cardiolipin deficiency activates the ISR in T_reg_ cells through a multifaceted mitochondrial dysregulation

**Fig. 5:**
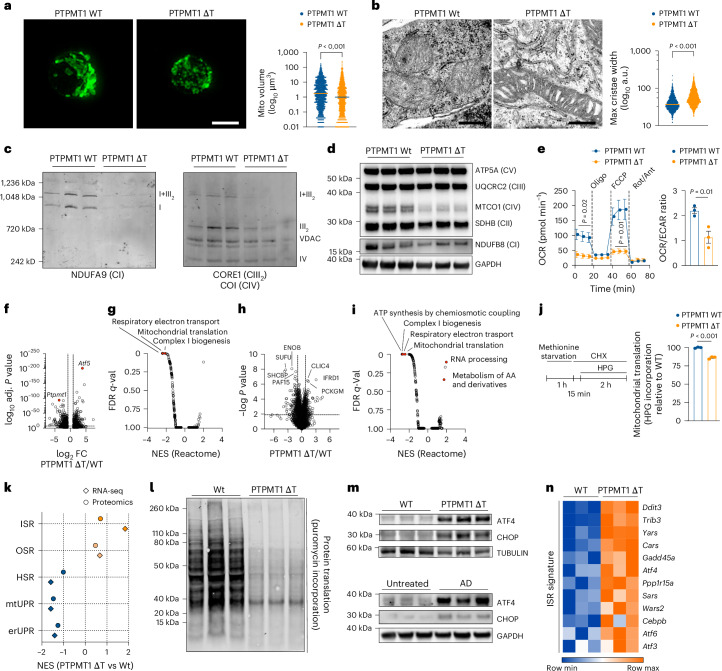
Cardiolipin deficiency activates ISR in T_reg_ cells through a multifaceted mitochondrial dysregulation. **a**, Representative confocal microscopy images showing mitochondria (TOM20) and mitochondrial volume quantification (right) from in vitro generated PTPMT1 WT and PTPMT1 ΔT T_reg_ cells (*N* = 1,653 mito in PTPMT1 WT and *N* = 1,983 in PTPMT1 ΔT over three independent experiments). Scale bar, 5 μm. **b**, Representative electron microscopy images (left) and cristae width quantification (right) from in vitro generated PTPMT1 WT and PTPMT1 ΔT T_reg_ cells (*N* = 1,271 cristae in PTPMT1 WT and *N* = 1,243 in PTPMT1 ΔT over three independent experiments). Scale bars, 500 nm. a.u., arbitrary units. **c**, Blue Native PAGE analysis of in vitro generated PTPMT1 WT and PTPMT1 ΔT T_reg_ cells. Representative of three independent experiments. **d**, Immunoblot analysis of in vitro generated PTPMT1 WT and PTPMT1 ΔT T_reg_ cells. Representative of three independent experiments. **e**, OCR at basal level and following oligomycin (Oligo), fluoro-carbonyl cyanide phenylhydrazone (FCCP) and rotenone + antimycin A (Rot/Ant) addition (left) and OCR/ECAR ratio (right). **f**, Volcano plot of differentially expressed genes (DEGs) between PTPMT1 WT and PTPMT1 ΔT T_reg_ cells. Negative log_10_ adjusted *P* values are plotted against the log_2_-fold change. Selected genes are labelled. *P* values were calculated using Wald test and adjusted *P* values were calculated using the Benjamini–Hochberg approach, with the DESeq2 package. **g**, Volcano plot illustrating pathway enrichment analysis (gene-set enrichment analysis) of the transcriptomic profile of PTPMT1 WT and PTPMT1 ΔT T_reg_ cells. False discovery rate (FDR) *q* value calculated as described in the Methods. **h**, Volcano plot of differentially expressed proteins between PTPMT1 WT and PTPMT1 ΔT T_reg_ cells. Selected proteins are labelled. *P* values were calculated using an unpaired two-tailed Student’s *t*-test. **i**, Volcano plot illustrating pathway enrichment analysis (gene-set enrichment analysis) on the proteomic profile of PTPMT1 WT and PTPMT1 ΔT T_reg_ cells. FDR *q* value was calculated as described in the Methods. **j**, Mitochondrial translation in PTPMT1 WT and PTPMT1 ΔT T_reg_ cells. **k**, Enrichment analysis on indicated pathways from RNA-seq and proteomics datasets of PTPMT1 WT and PTPMT1 ΔT T_reg_ cells. **l**, Total protein translation in PTPMT1 WT and PTPMT1 ΔT T_reg_ cells. Representative of three independent experiments. **m**, Protein expression of ATF4 and CHOP in PTPMT1 WT and PTPMT1 ΔT T_reg_ cells (upper) and alexidine dihydrochloride (AD)-treated WT cells (lower). Representative of three independent experiments. **n**, mRNA expression of selected genes of the ISR signature in PTPMT1 WT and PTPMT1 ΔT T_reg_ cells. Data are shown as the mean ± s.e.m. of three independent experiments unless indicated. Statistical comparisons between two groups were calculated using an unpaired two-tailed Student’s *t*-test. Exact *P* values are indicated. AA, amino acids. Source data

**Extended Data Fig. 5:**
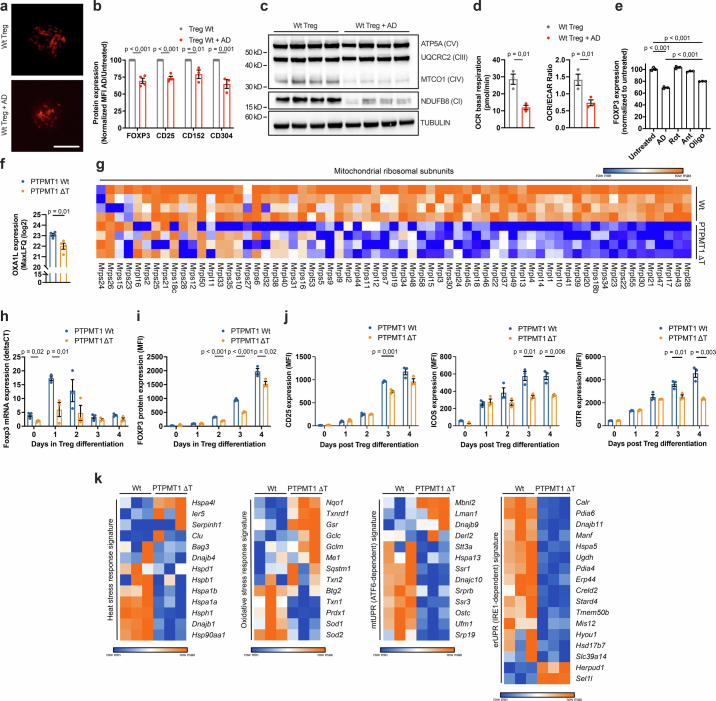
Cardiolipin deficiency in Treg cells promotes metabolic, transcriptional and proteomic remodeling. (**a**) Representative confocal microscopy images showing mitochondria (TOM20) in Wt Treg cells treated with DMSO or 1μM AD for the last 24 h of Treg differentiation. Scale bar 5 μm. (**b**) Expression of FOXP3, CD25, CD152 and CD304 protein levels in Wt Treg cells treated with DMSO or 1μM AD for the last 24 h of Treg differentiation. (**c**) Immunoblot of Wt Treg cells treated with DMSO or 1μM AD for the last 24 h of Treg differentiation. Representative experiment out of three. (**d**) Basal OCR (left) and OCR/ECAR ratio (right) of Wt Treg cells treated with DMSO or 1μM AD for the last 24 h of Treg differentiation. (**e**) FOXP3 expression upon 6h treatment with the indicated drugs (**f**) Expression of OXA1L in PTPMT1 Wt and PTPMT1 ΔT CD4^+^ cells. (**g**) Expression of mitochondrial ribosomal subunits PTPMT1 Wt and PTPMT1 ΔT CD4^+^ cells. (**h**) *Foxp3* mRNA expression during Treg differentiation in PTPMT1 Wt and PTPMT1 ΔT CD4^+^ cells. (**i**) FOXP3 protein expression during Treg differentiation in PTPMT1 Wt and PTPMT1 ΔT CD4^+^ cells. (**j**) CD25, ICOS, and GITR protein expression during Treg differentiation in PTPMT1 Wt and PTPMT1 ΔT CD4^+^ cells. (**k**) mRNA expression of selected genes of the indicated signatures in PTPMT1 Wt and PTPMT1 ΔT Treg cells. Data shown as mean ± SEM of three independent experiments unless indicated. Statistical comparisons between two groups were calculated using an unpaired two-tailed Student’s t test. Exact p-values are indicated. Source data

We next investigated how cardiolipin deficiency regulates T_reg_ function. The T_reg_ helper T cell subset has been suggested to rely on mitochondrial respiration more than other helper T cell subsets^24^. We therefore set out to test whether modulation of cardiolipin content could influence the mitochondrial function of T_reg_ cells. Confocal microscopy analysis of CD4^+^ T cells differentiated in vitro to T_reg_ cells showed a more fragmented mitochondrial morphology with reduced volume per mitochondrion in PTPMT1 ΔT compared to WT T_reg_ cells (Fig. 5a). A similar phenotype in mitochondrial fragmentation (as well as in the expression of key T_reg_ markers) was produced by the acute pharmacological inhibition of cardiolipin synthesis using the specific PTPMT1 inhibitor (alexidine dihydrochloride; Extended Data Fig. 5a,b). Mitochondrial ultrastructure analysed by electron microscopy over more than 1,000 individual cristae showed remodelled cristae with wider maximal cristae width in PTPMT1 ΔT cells (Fig. 5b). Cardiolipin interacts with complex I, III and IV to stabilize respiratory chain complexes in their quaternary structures called respiratory chain supercomplexes (RCSs) to improve electron transfer efficiency^53^. Mitochondria from PTPMT1 ΔT cells showed reduced RCS I + III_2_ when probed with antibodies targeting either complex I or complex III (Fig. 5c). Not surprisingly, RCSs containing complex IV were not observed as PTPMT1 WT and PTPMT1 ΔT mice are on the C57BL/6J background, which harbours a mutation in *SCAF1*, encoding a necessary protein for I + III_2_ + IV RCS formation^54^. We also found that NDUFB8 and MTCO1 (subunits of complex I and complex IV, respectively) were reduced in both PTPMT1 ΔT cells and alexidine dihydrochloride-treated WT T_reg_ cells (Fig. 5d and Extended Data Fig. 5c), potentially linked with the overall reduced levels of RCS. In line with these findings, we observed a lower oxygen consumption rate (OCR) and OCR/extracellular acidification rate (ECAR), implying lower oxidative phosphorylation in both PTPMT1 ΔT cells and alexidine dihydrochloride-treated WT T_reg_ cells (Fig. 5e and Extended Data Fig. 5d). Acute cardiolipin synthesis inhibition had a more pronounced effect on FOXP3 expression than the acute inhibition of single respiratory chain complexes (Extended Data Fig. 5e), supporting the hypothesis that, while T_reg_ cells are not exclusively regulated by cardiolipin, they demonstrate particular sensitivity to cardiolipin deficiency that cannot be fully explained by generalized respiratory dysfunction alone. The analysis of the transcriptional and proteomic profile of PTPMT1 WT and PTPMT1 ΔT_reg_ cells suggested that cardiolipin deficiency could also trigger impaired mitochondrial translation (Fig. 5f–i). To test this hypothesis, we measured mitochondrial translation by specifically inhibiting cytoplasmic translation with cycloheximide and incubating the cells for 2 h with the methionine alkyne analog L-homopropargylglycine (L-HPG) after 1 h of methionine starvation (Fig. 5j). HPG incorporation was detected using a click chemistry approach conjugating the alkyne HPG with an Azide AF488 followed by FACS analysis. This experiment showed that mitochondrial translation is reduced in PTPMT1 ΔT_reg_ cells (Fig. 5j). In line with that, cardiolipin-deficient cells showed a reduced amount of OXA1L, critical for membrane docking of mitochondrial ribosomes^37^, as well as the profound downregualtion of the overall mitochondrial ribosomal subunits profile (Extended Data Fig. 5f,g).

This metabolic complex dysregulation was signalled back to the nucleus, leading to alteration in the overall transcriptome (Fig. 5f,g) including downregulation of transcription and then translation of the key T_reg_ transcription factor FOXP3 (Extended Data Fig. 5h,i), as well as its downstream proteins, such as CD25, ICOS and GITR (Extended Data Fig. 5j) during T_reg_ differentiation.

To define the nature of the mitonuclear retrograde signalling triggered by cardiolipin deficiency, we examined different stress signalling pathways activated in response to mitochondrial insults: the ISR, the heat stress response, the oxidative stress response, the ATF6-dependent mitochondrial UPR and the IRE1-dependent erUPR^55^. Enrichment scores calculated on both the transcriptional and proteomic profiles of PTPMT1 WT and PTPMT1 ΔT_reg_ cells showed the ISR to be prominently active in PTPMT1 ΔT_reg_ cells compared to the other stress pathways (Fig. 5k and Extended Data Fig. 5k). The ISR triggers inhibition of cap-dependent translation with the upregulation of an ATF4-dependent and CHOP-dependent subset of genes required for the stress response^46^. In line with this, we observed reduced overall protein translation (Fig. 5l) and upregulation of ATF4 and CHOP (encoded by the genes *Atf4* and *Ddit3*), both at transcriptional and protein levels together with their target genes (Fig. 5m,n). Moreover, *Atf5*, a direct target and interactor of ATF4 and CHOP^56^, was the most significantly upregulated gene in PTPMT1 ΔT_reg_ cells in our transcriptomic analysis (Fig. 5f).

### Blockade of the ISR rescues PTPMT1 ΔT lifespan by restoring T_reg_ profile

**Fig. 6:**
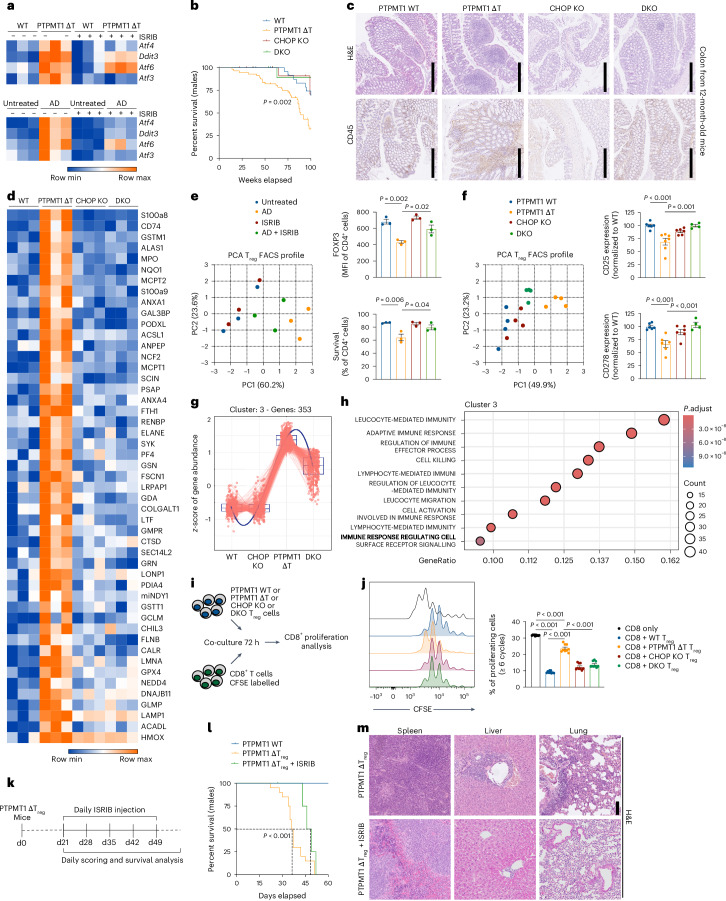
Blockade of ISR rescues PTPMT1 ΔT mice lifespan by restoring T_reg_ profile. **a**, mRNA expression of indicated genes in PTPMT1 WT and PTPMT1 ΔT T_reg_ cells (upper) and alexidine dihydrochloride-treated WT cells (lower) following ISRIB treatment. **b**, Kaplan–Meier survival curve of PTPMT1 WT (*N* = 25) and PTPMT1 ΔT (*N* = 57). CHOP KO (*N* = 12) and PTPMT1 ΔT CHOP DKO (*N* = 14) male mice. **c**, Representative histological images of colon sections from 12-month-old PTPMT1 WT, PTPMT1 ΔT, CHOP KO and PTPMT1 ΔT CHOP DKO mice stained with H&E and immunostained for CD45. Scale bars, 500 μm. Representative of at least three biological replicates per group. **d**, Protein expression of CHOP-dependent ISR markers in PTPMT1 WT, PTPMT1 ΔT, CHOP KO and PTPMT1 ΔT CHOP DKO T_reg_ cells. **e**, Principal component analysis (left), FOXP3 expression and survival (right) of untreated and alexidine dihydrochloride-treated WT T_reg_ cells following ISRIB treatment. **f**, Principal component analysis (left, *N* = 4 per genotype), CD25 (PTPMT1 WT *N* = 6, PTPMT1 ΔT *N* = 7, CHOP KO *N* = 6 and PTPMT1 ΔT CHOP DKO *N* = 4) and CD278 (PTPMT1 WT *N* = 6, PTPMT1 ΔT *N* = 6, CHOP KO *N* = 6 and PTPMT1 ΔT CHOP DKO *N* = 4) expression in T_reg_ cells (right). **g**, Unbiased clustering analysis of transcriptomic profile of PTPMT1 WT (*N* = 3), PTPMT1 ΔT (*N* = 3), CHOP KO (*N* = 2) and PTPMT1 ΔT CHOP DKO (*N* = 2) T_reg_ cells (cluster 3). Box plots report the 25% (lower hinge), median (centre line) and 75% (upper hinge) quantiles. Whiskers indicate observations equal to or outside the hinge ± 1.5 times the interquartile range (IQR). Outliers (beyond 1.5 times the IQR) are not plotted. **h**, Pathway enrichment analysis of genes included in cluster 3. **i**, Schematic experimental design of T_reg_ cell suppression assay. **j**, Frequencies of CD8^+^ T cells reaching at least division 6 in in vitro suppression assay (*N* = 9 per group). **k**, Schematic experimental design for in vivo ISRIB treatment. **l**, Kaplan–Meier survival curve of PTPMT1 WT, PTPMT1 ΔT, PTPMT1 ΔT + ISRIB groups. **m**, Representative H&E images of spleens, livers and lungs of mice treated as indicated. Representative of at least three biological replicates per group. Scale bar, 100μm. Data are shown as the mean ± s.e.m. of three independent experiments unless indicated. Statistical comparisons between two groups were calculated by using an unpaired two-tailed Student’s *t*-test. Statistical comparison among more than two groups were calculated using one-way ANOVA with Tukey’s correction. Log-rank (Mantel–Cox) test was performed to compare survival curves in **b** and **l**. Exact *P* values are indicated. Source data

**Extended Data Fig. 6:**
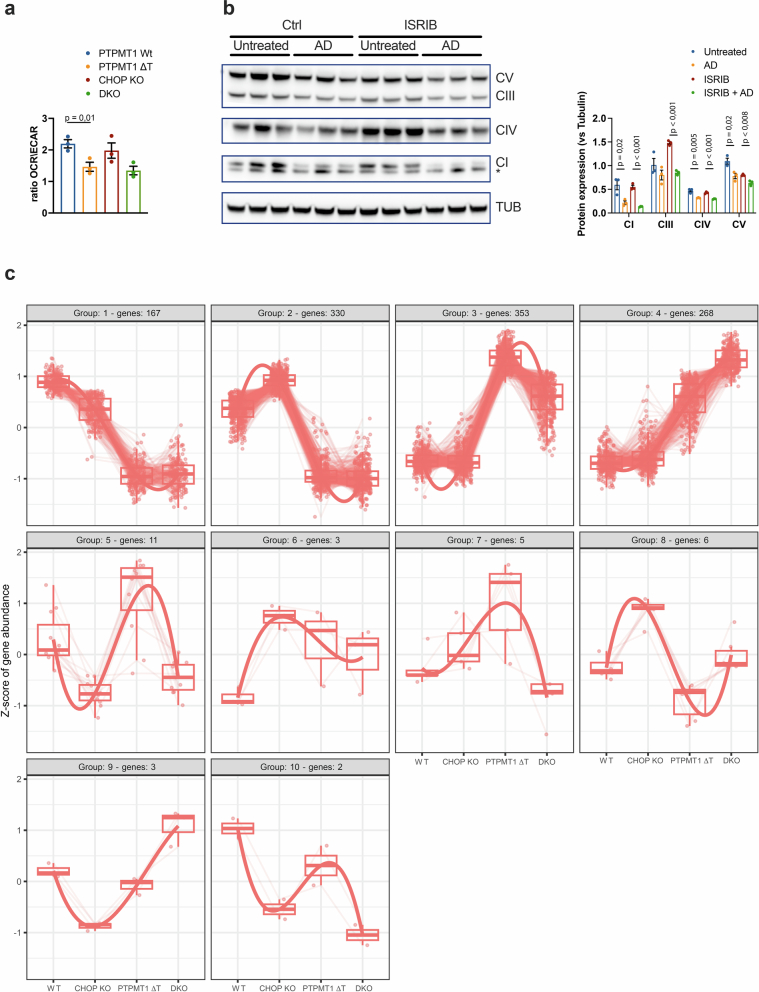
ISR inhibition in PTPMT1 ΔT Treg cells restores immune transcriptional profile without improving metabolic fitness. (**a**) OCR/ECAR ratio in Treg cells of the indicated genotypes. (**b**) Protein expression of the indicated proteins in Untreated and AD-treated Treg cells ± ISRIB. (**c**) Cluster analysis of transcriptomic profile of Treg cells of the indicated genotypes. Data shown as mean ± SEM of three independent experiments unless indicated. All boxplots report the 25% (lower hinge), median (centre line) and 75% (upper hinge) quantiles. Whiskers indicate observations equal to or outside the hinge ± 1.5× the interquartile range (IQR). Outliers (beyond 1.5× the IQR) are not plotted. Data shown as mean ± SEM of three independent experiments unless indicated. Statistical comparison among more than two groups were calculated using the Kruskall-Wallis test with Tukey’s correction. Exact p-values are indicated. Source data

In culture, the ISR in PTPMT1 ΔT_reg_ and alexidine dihydrochloride-treated cells could be reverted to WT levels with the small-molecule ISR inhibitor (ISRIB; Fig. 6a). To evaluate the in vivo effect of blocking the ISR in PTPMT1 ΔT mice, we generated PTPMT1 ΔT CHOP DKO mice. Of note, PTPMT1 ΔT CHOP DKO rescued the lifespan defect of PTPMT1 ΔT mice (Fig. 6b). Moreover, histopathology of small intestine/colon of PTPMT1 ΔT CHOP DKO mice showed an improved phenotype with preserved structure and reduced immune infiltration (Fig. 6c). Analysis of the proteomic profiles of WT, PTPMT1 ΔT, CHOP KO and PTPMT1 ΔT CHOP DKO T_reg_ cells revealed the CHOP-dependent ISR signature reversal (Fig. 6d). We hypothesized the improved phenotype in PTPMT1 ΔT CHOP DKO mice could be mediated by a rescue in T_reg_ profile. To test this hypothesis, we analysed cardiolipin-deficient T_reg_ cells following ISR inhibition. Both ISRIB treatment in alexidine dihydrochloride-mediated cardiolipin-deficient T cells and CHOP KO in PTPMT1 ΔT_reg_ cells were able to restore, although not completely, T_reg_ profile (Fig. 6e,f). This partial rescue is not due to a recovery of mitochondrial function but to the inhibition of detrimental downstream transcriptional modules that affect T_reg_ cells. In particular, we previously observed that cardiolipin-deficient cells present lower oxygen consumption and lower OCR/ECAR ratio (Fig. 5e), which was not reverted in PTPMT1 ΔT CHOP DKO T_reg_ cells (Extended Data Fig. 6a). Moreover, ISR inhibition in cardiolipin-deficient cells did not rescue levels of respiratory chain subunits (Extended Data Fig. 6b). To gain a broader insight into the regulatory circuits affected by CHOP deficiency in PTPMT1 ΔT CHOP DKO T_reg_ cells, we turned to an unbiased clustering analysis of the transcriptomic profile of WT, PTPMT1 ΔT, CHOP KO and PTPMT1 ΔT CHOP DKO T_reg_ cells. Our analysis reported nine clusters (Extended Data Fig. 6c). Cluster 3 (containing 353 genes) was of particular interest showing genes upregulated in PTPMT1 ΔT T_reg_ cells partially returning towards WT baseline in PTPMT1 ΔT CHOP DKO T_reg_ cells (Fig. 6g). Of note, pathway enrichment analysis showed these genes were involved almost exclusively in immune-related pathways (Fig. 6h). These data suggest that ISR inhibition in PTPMT1 ΔT CHOP DKO T_reg_ cells restores T_reg_ transcriptional profile. To test whether this was sufficient to restore immunosuppressive function, we performed a suppression assay. PTPMT1 ΔT CHOP DKO T_reg_ cells were able to suppress CD8^+^ T cell proliferation to a much greater extent than PTPMT1 ΔT T_reg_ cells (Fig. 6i,j). This confirms ISR inhibition in PTPMT1 ΔT T_reg_ cells improves T_reg_ cell function by restoring the immune transcriptional profile of T_reg_ cells, even without a rescue in their metabolic properties.

Finally, we investigated whether inhibition of the ISR could also benefit the more severe phenotype of PTPMT1 ΔT_reg_ mice. To do so, we designed an experiment in which PTPMT1 ΔT_reg_ mice were pharmacologically treated daily with ISRIB, previously used for similar purposes^47,48^. In brief, PTPMT1 ΔT_reg_ mice were injected intraperitoneally with ISRIB (5 mg per kg body weight per day) for 4 weeks starting at weaning (21 days of age; Fig. 6k). Of note, ISRIB treatment delayed the onset of autoimmune symptoms and extended median lifespan, a phenotype consistent with a partial rescue of T_reg_ cell function (Fig. 6l). This was also associated with an improved histopathology of multiple organs including spleen, liver and lung collected when mice were 6 weeks old (Fig. 6m).

Overall, our analysis reveals that ISR is a pharmacologically and genetically targetable maladaptive stress signalling pathway activated in response to cardiolipin deficiency in T cells.

### Inflammatory and T_reg_ alterations in Barth syndrome mouse models and patients

**Fig. 7:**
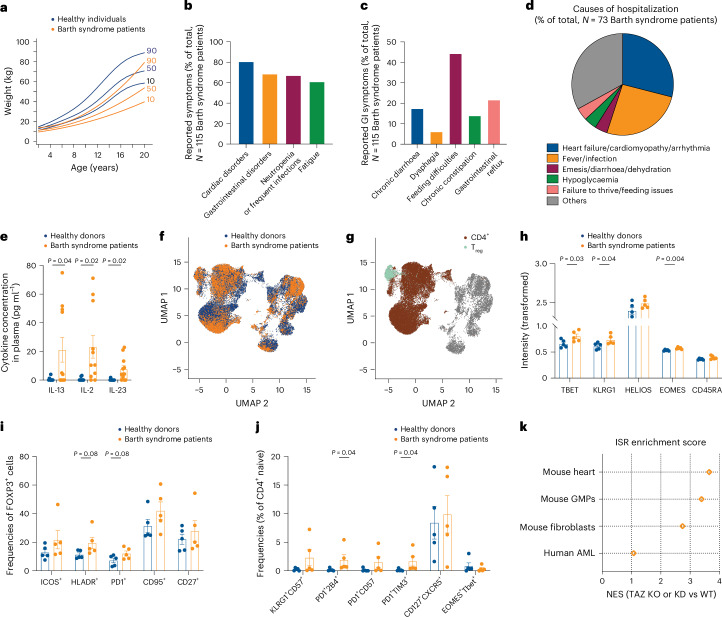
Inflammatory profile and ISR signature in individuals with Barth syndrome. **a**, Weight for age centile distribution in individuals with Barth syndrome versus general US male population. **b**, Frequencies of reported symptoms in a cohort of individuals with Barth syndrome (*N* = 115). **c**, Frequencies of reported gastrointestinal symptoms in a cohort of individuals with Barth syndrome (*N* = 115). **d**, Frequencies of causes of hospitalization in a Barth syndrome cohort (*N* = 73). **e**, IL-13, IL-2 and IL-23 cytokine concentration in plasma from healthy donors (*N* = 8) and individuals with Barth syndrome (*N* = 11). **f**, UMAP visualization plot of human PBMCs collected from healthy donors (*N* = 5) and individuals with Barth syndrome (*N* = 5) and analysed via high-dimensional spectral cytometry. **g**, UMAP visualization plot of human PBMCs collected from healthy donors (*N* = 5) and individuals with Barth syndrome (*N* = 5) highlighting CD4^+^ T cell and T_reg_ clusters analysed via high-dimensional spectral cytometry. **h**, Expression levels of the indicated proteins in the T_reg_ cell cluster from healthy donors (*N* = 5) and individuals with Barth syndrome (*N* = 5) samples. **i**, Frequencies of FOXP3^+^ T_reg_ cells co-expressing the indicated proteins in samples from healthy donors (*N* = 5) and individuals with Barth syndrome (*N* = 5). **j**, Frequencies of CD4^+^ cells co-expressing the indicated proteins in samples from healthy donors (*N* = 5) and individuals with Barth syndrome (*N* = 5). **k**, ISR normalized enrichment score (NES) calculated on publicly available datasets featuring the indicated TAZ KO and WT mouse and human tissues or cells. Data are shown as the mean ± s.e.m. of three independent experiments unless indicated. Statistical comparisons between two groups were calculated by using an unpaired one-tailed (**j**) or two-tailed (all other panels) Student’s *t*-test. Exact *P* values are indicated. GMP, granulocyte monocyte progenitor; AML, acute myeloid leukemia. Source data

**Extended Data Fig. 7:**
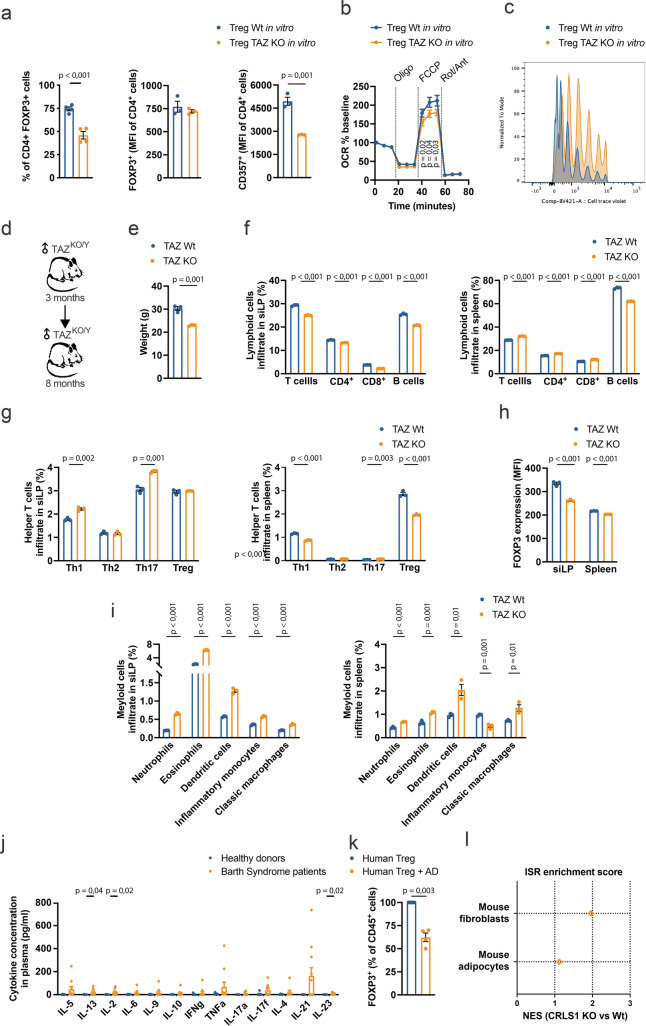
TAFAZZIN loss promotes inflammation in Barth syndrome mouse models and patients. (**a**) Frequencies of FOXP3^+^ expressing CD4^+^ cells (Wt N=4, TAZ KO N=4) (left panel), FOXP3 (Wt N=3, TAZ KO N=3) and CD357 cells (Wt N=3, TAZ KO N=3) expression (center and right panel) after 4 days in Treg differentiation conditions. (**b**) OCR in Wt and TAZ KO CD4^+^ cells after 4 days in Treg differentiation conditions. (**c**) Proliferation of Wt and TAZ KO CD4^+^ cells after 4 days in Treg differentiation conditions. (**d**) Schematic depiction of ageing TAZ KO mice used for *in vivo* immunophenotyping. (**e**) Weigh of 8 months old Wt and TAZ KO mice. (**f**) Lymphoid cell frequencies in siLP (left) and spleen (right) of 8 months old Wt and TAZ KO mice. (**g**) CD4^+^ Helper T cell subsets in siLP (left) and spleen (right) of 8 months old Wt and TAZ KO mice. (**h**) FOXP3 expression (as MFI) of FOXP3 expressing CD4^+^ T cells in siLP and spleen of 8 months old Wt and TAZ KO mice. (**i**) Myeloid cell frequencies in siLP (left) and spleen (right) of 8 months old Wt and TAZ KO mice. (**j**) Cytokine concentrations in plasma from healthy donors (N=8) and Barth syndrome patients (N=11). (**k**) Frequencies of live hCD4^+^ cells expressing FOXP3 after 4 days of Treg differentiation treated with DMSO (N=4) or 1 µM AD (N=4). (**l**) ISR enrichment score calculated on publicly available datasets featuring the indicated CRLS1 KO and WT mouse cells. Data shown as mean ± SEM of three independent experiments unless indicated. Statistical comparisons between two groups were calculated by using an unpaired two-tailed Student’s t test. Exact p-values are indicated. Source data

Cardiolipin deficiency in humans leads to Barth syndrome, an IEM caused by mutations in the *TAFAZZIN* gene, which encodes a transacylase required for cardiolipin remodelling^39–41^. We previously reported mild lymphopenia in patients with Barth syndrome^43^. Additionally, these patients are also characterized by neutropenia and recurrent infections^42^. Clinical reports also describe sporadic episodes of vomiting and diarrhoea^44^, as well as higher levels of the pro-inflammatory cytokine IL-6 (ref. ^45^) and excessive neutrophil activation^57^. These observations suggest that inflammation might play a role in the pathogenesis and prognosis of Barth syndrome. To test whether T_reg_ cell defects and gut inflammation could be features of TAFAZZIN (TAZ) KO animals (a mouse model for Barth syndrome) similarly to PTPMT1 ΔT mice, we analysed TAZ KO mice profiling in in vitro differentiated CD4^+^ T_reg_ cells and in vivo immunophenotyping spleens and siLP from 8-month-old TAZ KO mice. First, our analysis showed that TAZ KO CD4^+^ cells are functionally affected with reduced ability to differentiate in vitro into T_reg_ cells (Extended Data Fig. 7a). When cultured in vitro in a T_reg_ polarizing medium, TAZ CD4^+^ showed a differentiation defect as only around 40% of CD4^+^ cells expressed FOXP3 compared to around 75% of the WT CD4^+^ cells. The level of FOXP3 expression (FOXP3 mean fluorescence intensity or MFI) did not differ in the subset of CD4^+^ cells expressing FOXP3 between WT and TAZ KO cells, while the expression of additional functional markers like CD357 was reduced in TAZ KO cells (Extended Data Fig. 7a). TAZ KO T_reg_ cells also presented a metabolic impairment with reduced maximal respiration measured by Seahorse (Extended Data Fig. 7b) and a proliferation defect (Extended Data Fig. 7c) compared to WT T_reg_ cells. In addition, we analysed the in vivo immune phenotype of 8-month-old WT and TAZ KO mice (Extended Data Fig. 7d). In line with previous observations, TAZ KO mice were smaller compared to WT mice (22 g versus 30 g on average; Extended Data Fig. 7e) and presented a mild lymphopenia, especially evident in siLP tissue-resident T cells and B cells but also present in the spleen limited to B cells (Extended Data Fig. 7f). A more granular analysis of helper T cell subsets showed higher frequencies of T_H_1 and T_H_17 cells (expressing TBET and RORγt, respectively) in TAZ KO siLP (Extended Data Fig. 7g). FOXP3-expressing CD4^+^ T cells were present in similar frequencies in siLP but were less abundant in the spleens of TAZ KO mice (Extended Data Fig. 7g). FOXP3 expression was nevertheless significantly lower in both organs in TAZ KO mice (Extended Data Fig. 7h). This lymphoid phenotype was associated with accumulation of pro-inflammatory myeloid cells in siLP and spleens of TAZ KO mice including neutrophils, eosinophils, dendritic cells, inflammatory monocytes and classic macrophages (Extended Data Fig. 7i). Overall, this immunophenotyping suggests that T_reg_ impairment and inflammation are also features of TAZ KO mice. To gain further insight on the possible gastrointestinal and inflammatory profile of patients with Barth syndrome, we interrogated the Barth Syndrome Registry, a dataset of medical information on 115 living affected individuals in a 20-year follow-up study^58,59^. Data from the Barth Syndrome Registry show that individuals affected by Barth Syndrome are characterized by reduced weight for age percentile up to 20 years of age compared to healthy individuals (Fig. 7a). Interestingly, over 60% of individuals reported gastrointestinal disorders of different nature (Fig. 7b,c). In a subgroup of 73 individuals for whom further medical records were available, 62 were subjected to 250 hospitalizations during the study^55^. Fever/infection (26%), emesis/diarrhoea/dehydration (4.4%), hypoglycaemia (4%) and failure to thrive/feeding issues (4%) were reported as reasons for hospitalization in addition to the expected heart failure/cardiomyopathy/arrhythmia (29%; Fig. 7d). Moreover, among other less severe symptoms, 60% of individuals reported frequent cases of mouth ulcers^58^. Overall, these observations point to a possible inflammatory phenotype frequently affecting the oral tract and gastrointestinal system of at least a subset of individuals with Barth syndrome. To investigate this hypothesis further, we analysed plasma cytokine and peripheral blood mononuclear cell (PBMC) profiles of a small cohort of individuals with Barth syndrome. The quantification of plasma cytokine concentrations showed that the inflammation extended beyond the previously identified IL-6 to multiple other cytokines (Fig. 7e and Extended Data Fig. 7j). Interestingly, among the upregulated cytokines in individuals with Barth syndrome, IL-13 and IL-23 have been causally implicated in gut inflammation^60^ and could be linked to T_reg_ defects. The pharmacological inhibition of cardiolipin synthesis in healthy donor hCD4^+^ cells during T_reg_ differentiation resulted in lower FOXP3 expression (Extended Data Fig. 7k). Moreover, high-parametric flow cytometry analysis of PBMCs from individuals with Barth syndrome showed an altered T_reg_ profile compared to healthy donors (Fig. 7f,g). While the frequencies of T_reg_ (CD4^+^CD127^−^FOXP3^+^) cells were unchanged, their activation profile was markedly altered, with higher expression of TBET, KLRG1, EOMES, HLADR and PD-1 (Fig. 7h,i). This was accompanied by an activated and dysfunctional profile in the remaining non-T_reg_ CD4^+^ cells with indications of exhaustion/senescence, as suggested by the elevated expression of PD-1, 2B4 and TIM3 (Fig. 7j).

Finally, we interrogated multiple publicly available transcriptomic datasets from different TAFAZZIN-deficient mouse and human tissues to test whether the ISR was also active^61–64^. Notably, all datasets showed a positive enrichment for ISR (Fig. 7k). This was true also in datasets including cardiolipin synthase 1 (CRLS1) KO samples (Extended Data Fig. 7l)^37^. These observations confirm that, irrespective of the mechanism behind it, cardiolipin deficiency activates ISR. Altogether, we show that inflammation, altered T_reg_ cell function and an active ISR signature are present following TAZ deficiency. Moreover, our analysis highlights the possibility that they might also be clinical features of at least a subset of patients affected by Barth syndrome, a clinically relevant model of cardiolipin deficiency.

## Discussion

Host–microbiota interactions are crucial to maintain gut homeostasis. While both microbial and genetic factors have been linked to colitis, we show that a cardiolipin–ISR mitonuclear axis in T cells is key in maintaining tissue homeostasis. Alterations in this mitonuclear axis in T cells predispose to colitis by impairing tolerance to microbiota and sensitizing to pathobiont infections. Indeed, cardiolipin deficiency in PTPMT1 ΔT mice is sufficient to promote gut inflammation. This condition is already present in the absence of dysbiosis and then further deteriorates following pathobiont challenge.

Mitochondria control multiple T cell properties. They shape T cell differentiation, effector functions and long-term T cell memory^29^. Moreover, T cell-specific mitochondrial alterations might also critically contribute to ageing^50^. For example, TFAM deficiency in T cells results in CD4^+^ T cell skewing towards a T_H_1 phenotype, leading to systemic inflammation driven by excessive IFNγ and TNF secretion by T cells^50^. Of note, cardiolipin synthesis deficiency (mediated by either genetic or pharmacological inhibition of PTPMT1) does not promote T_H_1 skewing or transdifferentiation of other CD4^+^ T cell subsets to T_H_1 cells but it triggers T_reg_ defects. Our data suggest that T_reg_ cells with their unique lipidome profile (compared to other helper T cell subsets) are particularly sensitive to cardiolipin deficiency, which induces a multifaceted mitochondrial dysfunctional phenotype. The resulting maladaptive mitochondrial retrograde signalling further contributes to the impaired function of T_reg_ cells and its targeting might be a promising strategy to mitigate the extreme consequences of mitochondrial diseases. Indeed, our data suggest that ISR inhibition in PTPMT1 ΔT T_reg_ cells is sufficient to improve T_reg_ function by restoring their immune transcriptional profile, even without a rescue in their metabolic properties. Altogether, these observations suggest mitochondrial deficiencies in T cells have subset-specific and tissue-specific consequences. Indeed, despite that tissue-resident T_reg_ cells share a common tissue-residency programme^22^, the diverse spectrum of inflammation severity observed in different organs in PTPMT1 ΔT mice suggests that microenvironmental cues render some tissue permissive and some non-permissive for the inflammatory condition to manifest. Microenvironment imposes tissue-specific metabolic pressure on T_reg_ cells^65^, a pressure sustainable only up to a threshold level defined by each specific metabolic dysfunction. Our data suggest that the gut microenvironment represents a particularly challenging environment for cardiolipin-deficient T cells. This appears to be true irrespective of the mechanism leading to cardiolipin deficiency. Indeed, mice lacking the activity of the cardiolipin remodelling acyl-transferase TAFAZZIN present T_reg_ cell defects and signs of gut inflammation. In addition, intraepithelial CD8^+^ lymphocytes in the mouse gut also rely on TAFAZZIN for controlled activation and pathogen clearance^66^.

The systemic and gut-specific inflammation conserved in at least a subset of individuals with Barth syndrome may similarly stem (at least partially) from impaired T_reg_ cell function and the resulting activation of non-T_reg_ CD4^+^ T cells and neutrophils. Barth syndrome is classified as an IEM disorder^67^. This category encompasses pathologies caused by mutations in genes encoding enzymes, kinases, adaptors and transcription factors that govern metabolic pathways. Despite the critical role of metabolism in immunity, only a subset of IEM disorders has officially recognized immunological phenotypes and is clinically treated accordingly^29,43,68,69^. Our human data align with previous CRISPR screening results^70^ suggesting that the intersection between IEMs and inborn errors of immunity (IEIs)^71^ is underappreciated. This may be due to the prevalence of severe non-immunological features of IEM disorders, which obscure the immune phenotype, and the lack of a systematic analysis of immune features in IEMs. We hypothesize that a partially immunocompromised or inflammatory state driven by IEMs might critically contribute to the prognosis (and overall condition) of these patients making them, for example, more susceptible to some infections. Among these infections, the ones by pathobionts represent an interesting category. Studies on the global prevalence of *H. pylori* suggest almost half of the world population might be infected by this pathobiont^72^. Nevertheless, most infections go unnoticed as they do not manifest symptoms with only around 20% being symptomatic instead^73^. Genetic and environmental factors influence the pathogenicity of pathobionts, including the ones from the *Helicobacter* family, by acting on the tolerogenic mechanisms operating in the gut to control their presence^9^. Barth syndrome, as well as many other IEMs, shows a wide range of penetrance and severity. Some patients die from cardiac events during the first year of life, while others reach adult age. Of note, both cardiac events in the first year of life as well as a sudden deterioration of the condition in adults are often anticipated by infections^44^. In this perspective, pathobiont infections might find a more susceptible population in (at least a subset of) patients with IEMs and trigger colitis and a deterioration of the underlying metabolic pathology. Overall, mitochondrial deficiencies may be risk factors for gut inflammation and IBD by compromising T_reg_ cell function. This would extend beyond clinically recognized IEM disorders. Non-pathogenic single-nucleotide polymorphisms affecting mitochondrial metabolism may also partially impair T_reg_-mediated tolerance to microbiota, predisposing individuals to colitis and gut inflammation. We propose that these so far underappreciated immunopathological features observed in patients with IEMs, including Barth syndrome, should be more systematically considered and analysed in both scientific research and clinical practice.

In conclusion, we show that a cardiolipin–ISR mitonuclear axis controls host–microbiota interaction in the gut compromising tolerance to *Helicobacter* species in a T_reg_ cell-dependent manner. Moreover, we show that metabolic fitness of T cells in the gut governs the balance between tolerance and pathogenicity of gut microbiota potentially explaining the observed diversity in the clinical outcome of pathobiont infections in large populations studies. Finally, we propose that IEMs might predispose patients to colitis and gut inflammation by altering immune tolerance to microbiota.

## Methods

### Mice and immunizations

#### Mice

PTPMT1 floxed (RRID: IMSR_JAX: 020775) mice were purchased from The Jackson Laboratory. cGAS^−/−^ mice were generated in-house by ivRF CECAD and kindly donated by M. Pasparakis. CHOP^−/−^ (RRID: IMSR_JAX: 005530) were kindly donated by A. Trifunovic. PTPMT1 floxed mice were crossed to CD4-Cre mice as previously described^43^ or to Foxp3-YFP-Cre (RRID: IMSR_JAX: 016959) kindly donated by M. Beyer (DZNE). TAZ floxed mice^74^ were kindly donated by D. Strathdee (Cancer Research UK Beatson Institute) and crossed in-house with CMV-Cre mice (RRID: IMSR_JAX:006054) to generate TAZ KO mice. Mice were maintained at the ivRF CECAD Research Institute (ivRF A) or Max Planck Institute of Immunobiology and Epigenetics (ivRF B) under specific-pathogen-free conditions and cared for according to the Institutional Animal Use and Care Guidelines and under a 12-h dark–12-h light cycle in individually ventilated cages (Greenline GM500, Tecniplast) at 22 °C ± 2 °C and a relative humidity of 55% ± 5%. All mice had unlimited access to water and fed a standard chow diet (Sniff, V1554-300 or V1185-300) ad libitum. The health monitoring programme followed FELASA recommendations. Briefly, germ-free NMRI mice are used as bedding sentinels receiving dirty bedding from up to 100 other cages weekly for 3 months before being tested for all pathogens and opportunists recommended in the guidelines. Breeding of the animals was approved by Landesamt für Natur, Umwelt und Verbraucherschutz Nordrhein-Westfalen (LANUV NRW). EAE, *L. monocytogenes*, CD4^+^ T transfer studies and ISRIB treatment were approved either by the Regierungsprasidium Freiburg or by LANUV NRW. *H. hepaticus* infection was conducted at University of Oxford in accordance with the UK Scientific Procedures Act of 1986, and by persons holding a personal license. The project license governing the mouse studies was reviewed by the University of Oxford’s Animal Welfare and Ethical Review Board and approved by the Home Office of His Majesty’s Government. All mice were used for experiments between 9 and 12 weeks of age or at 12 months of age unless otherwise indicated. For *L. monocytogenes* and *H. hepaticus* experiments male mice were used, for EAE female mice were used, and for in vitro studies both sexes were used interchangeably (no influence or association of sex on the results was observed). Animals were randomly assigned to experimental group, and cages contained mice of all different experimental groups.

#### Human cells

Fresh buffy coats from healthy donors were kindly provided by the Uniklinik Köln under approval by the ethics committee. PBMCs from healthy donors and patients with Barth syndrome were collected at University of Bristol in accordance with the Helsinki Declaration with approval from the UK NHS Research Ethics committee (permit number 09/H0202/52). Age and sex of healthy donors provided by the Uniklinik Köln were blinded to the authors. Patients with Barth syndrome were males (Barth syndrome group average age 13 years, minimum 6 years, maximum 28 years; healthy donor group average age 28 years, minimum 22 years, maximum 39 years). Informed consent was provided by all patients, or by their parents, in the case of children. Human CD4^+^ T cells were isolated using CD4^+^ T cell kits (Stem Cell Technologies, 17952) and treated with DMSO or alexidine dihydrochloride (1 μM). Researchers were blinded to the identity of the donors, and age or sex matching was not performed. Sample size is indicated in the figure legends.

#### *H. hepaticus* culture and oral gavage

*H. hepaticus* NCI-Frederick isolate 1A (strain 51449) was grown on blood agar plates containing 7.5% laked horse blood (Thermo Scientific) and Skirrow Campylobacter supplement (Oxoid) under microaerophilic conditions at 37 °C with agitation. Cultures were expanded for 48 h in Tryptone Soy Broth (Fisher) containing 10% FCS (Gibco) and the above antibiotics. The concentration of bacteria was determined by optical density (OD) analysis at 600 nm. Mice were fed 1 × 10^8^ colony-forming units of *H. hepaticus* (equivalent to 1 OD unit) by oral gavage using a curved 22-gauge needle for a total of two doses 24 h apart.

#### *L. monocytogenes* immunization

Age- and sex-matched mice were injected intravenously (i.v.) as previously described^43^ with a sublethal dose of 1 × 10^6^ colony-forming units of recombinant *L. monocytogenes* expressing OVA deleted for actA (LmOVA ΔActa) for primary immunizations.

#### Mouse EAE model

EAE was induced by immunizing mice subcutaneously with 200 μg of myelin oligodendrocyte glycoprotein peptide (MOG35–55: MEVGWYRSPFSRVVHLYRNGK) emulsified in complete Freund’s adjuvant (supplemented with killed *Mycobacterium tuberculosis* strain H37RLa) and intraperitoneal injections of 200 ng pertussis toxin (Hooke Labs) at the time of immunization and 24 h later. The disease was scored daily on a scale of 0–5 as follows: 0, no overt signs of disease; 1, limp tail; 2, limp tail plus hindlimb weakness; 3, hindlimb paralysis; 4, hindlimb and forelimb paralysis; 5, moribund. At the end of experiment, the mice were euthanized for analysis of the T cells infiltrated into the brain and spinal cord. Mice that did not develop symptoms of EAE were not excluded from the analysis.

#### T cell transfer colitis model

For experiments involving the co-transfer of naive CD4^+^ T cells with T_reg_ cells, we adoptively transferred into 12-week-old *Rag2*^−*/*−^ recipient mice (Jackson) by intravenous injection 4 × 10^5^ naive CD4^+^ T cells (CD45Rb^hi^CD25^−^CD44^lo^CD62L^+^) from CD45.1 mice with 1.5 × 10^5^ T_reg_ cells (day 4 after CD4^+^ T cell isolation and T_reg_ cell differentiation) from either PTPMT1 ΔT mice or their WT littermate controls.

#### In vivo ISRIB treatment

Mice were administered 5 mg per kg per day ISRIB (SIGMA), dissolved with heat in 40% saline, 50% polyethylene glycol, 10% DMSO or vehicle alone intraperitoneally daily for 4 weeks.

### Mouse cell isolation and culture

#### Isolation of lymphocytes from the spleen and mLNs

Spleens and lymph nodes were collected from 8–12-week-old mice and mashed with a syringe plunger in a Petri dish with 5 ml of T cell medium (TCM; 1640 RPMI with 10% FCS, 4 mM L-glutamine, 1% penicillin–streptomycin, 55 mM β-mercaptoethanol). The cell suspension was filtered through a 70-μm strainer, centrifuged at 400*g* for 5 min at 4 °C and resuspended in 1 ml of LCK (Thermo Fisher Scientific, A1049201) to lyse red blood cells. After 3 min TCM was added, and the cell suspension was centrifuged at 400*g* for 5 min at 4 °C. The pellet was resuspended in 1 ml of TCM and filtered through 40-μm cell strainers.

#### Isolation of lymphocytes from the small intestine

Following collection, fat tissue bordering the small intestine and Peyer’s patches was removed. Small intestines were cut open, washed with ice-cold 1× PBS + 25 mM HEPES (Sigma-Aldrich, 83264-100ML-F), cut into 2-cm-long pieces and collected in medium containing RPMI 1640 (Thermo Fisher Scientific, 21875158), 25 mM HEPES (Sigma-Aldrich, 83264-100ML-F), 3% FCS (Gibco, 26140079), 4 mM glutamine (Life Technologies, 25030-024), 1% penicillin–streptomycin (Life Technologies, 15140-122) and 55 μM β-mercaptoethanol (Thermo Fisher Scientific, 21985023; hereafter named 3% FCS medium). Small intestines were incubated in 3% FCS medium supplemented with 5 mM EDTA (Thermo Fisher Scientific, 15575020) and 1 mM dithiothreitol (DTT; Sigma-Aldrich, 10197777001) for 25 min, at 37 °C, 5% CO_2_ and agitation (first incubation). After the incubation, cells in suspension were collected, consecutively strained through 100-μm and 40-μm strainers, washed and kept as the intraepithelial lymphocyte (IEL) fraction. The leftover small intestine pieces were thoroughly washed (four times) by shaking with ice-cold medium containing RPMI 1640, 25 mM HEPES (Sigma-Aldrich, 83264-100ML-F), 4 mM glutamine, 1% penicillin–streptomycin and 55 μM β-mercaptoethanol (hereafter named serum-free medium) added with 2 mM EDTA. Washed small intestine pieces were then finely chopped and incubated in serum-free medium added with 100 μg ml^−1^ Liberase TL (Roche, 05401020001) and 50 μg ml^−1^ DNase I (Sigma-Aldrich, 10104159001) for 35 min, at 37 °C, 5% CO_2_ and agitation (second incubation). After digestion, suspensions were strained through 70-μm strainers, washed and kept as the lamina propria fraction. Lamina propria and IELs were enriched for leucocytes using a three-layered Percoll (Merck, 17089101) gradient (Percoll 75%, 40% and 30%), centrifuged for 20 min at room temperature, at 680*g*, without any centrifuge acceleration and brake. The leucocyte layer between Percoll 75% and 40% was collected as the enriched lamina propria or IEL fraction, extensively washed and suspensions used for downstream applications.

#### Isolation of lymphocytes from the colon

Colons were isolated, cleaned, cut into small pieces, washed twice in RPMI 5% FCS supplemented with 5 mM EDTA and then once in RPMI 5% FCS containing 15 mM HEPES in a shaking incubator at 200 rpm for 20 min at 37 °C (IECs). Tissue digestion was performed in RPMI 5% FCS containing 1 mg ml^−1^ type VIII collagenase (Sigma-Aldrich, C2139-500MG) and 40 mg ml^−1^ DNase I (Sigma-Aldrich, 10104159001) at 37 °C for 60 min on a shaker at 200 rpm. Supernatants were then filtered on a 70-μm strainer, and a three-layered Percoll gradient (Percoll 75%, 40% and 30%) was used to obtain an enriched leucocyte fraction (layer between Percoll 75% and 40%).

#### Isolation of lymphocytes from liver, lung, kidney and brain

Whole liver was collected in ice-cold complete medium, finely chopped and incubated in RPMI 1640 supplemented with 1 mg ml^−1^ collagenase IV (Sigma-Aldrich, C2139-500MG) and 50 μg ml^−1^ DNase I (Sigma-Aldrich, 10104159001) for 45 min, at 37 °C, 5% CO_2_ and agitation. After digestion, suspensions were strained through 70-μm strainers, washed and enriched for leucocytes using Percoll 33% and centrifuging them for 20 min at room temperature, at 680*g*. The leucocyte pellet was collected, extensively washed, red blood cell lysis was performed and suspensions used for downstream applications. Lungs and kidneys were collected in ice-cold complete medium, finely chopped and incubated in complete medium added with 2 mg ml^−1^ collagenase IV (Sigma-Aldrich, C2139-500MG) and 50 μg ml^−1^ DNase I (Sigma-Aldrich, 10104159001) for 45 min, at 37 °C, 5% CO_2_ and agitation. Brains were collected in ice-cold complete medium, finely chopped and incubated in complete medium added with 2 mg ml^−1^ collagenase II (Sigma-Aldrich, C2139-500MG) and 50 μg ml^−1^ DNase I (Sigma-Aldrich, 10104159001) for 1 h, at 37 °C, 5% CO_2_ and agitation. After digestion, lung, kidney and brain suspensions were strained through 70-μm strainers, washed and enriched for leucocytes using a three-layer Percoll gradient (Percoll 75%, 40% and 30%), centrifuged for 20 min at room temperature, at 680*g*, without any centrifuge acceleration or brake. The leucocyte layer between Percoll 75% and 40% was collected, extensively washed and suspensions used for downstream applications.

#### CD4^+^ T cell isolation and differentiation

Lymphocytes were isolated from spleen and peripheral lymph nodes as described above. Purification of CD4^+^ T cells for cell culture was performed using the mouse CD4^+^ T cell isolation kit (Stem Cell Technologies, 19852), following the manufacturer’s instructions. Isolated CD4^+^ T cells (1 × 10^6^ per ml) were activated using a 48-well plate (Polycarbonate cell culture insert in multi-well plates; Falcon, 353078) coated with aCD3 (5 mg ml^−1^; Bio X Cell, BE0002) and soluble aCD28 (0.5 mg ml^−1^; Bio X Cell, BE0015-1) in TCM (described above) supplemented with 100 U ml^−1^ human recombinant IL-2 (10 ng ml^−1^; PeproTech, 200-02), TGF-β (10 ng ml^−1^; Thermo Fisher Scientific, 100-21-100UG), anti-IFNγ (4 μg ml^−1^; Bio X Cell, BP0055), anti-IL-4 (4μg ml^−1^; Bio X Cell, BP0045) to differentiate CD4^+^ T cells into T_reg_ cells. Cells were supplemented with 100 U ml^−1^ human recombinant IL-2 (10 ng ml^−1^; PeproTech, 200-02), IL-12 (10 ng ml^−1^; PeproTech, 210-12) and anti-IL-4 (4μg ml^−1^; Bio X Cell, BP0045) to differentiate CD4^+^ T cells into T_H_1 cells. Cells were supplemented with 100 U ml^−1^ human recombinant IL-2 (10 ng ml^−1^; PeproTech, 200-02), IL-4 (10 ng ml^−1^; PeproTech, 214-14) and anti-IFNγ (4 μg ml^−1^; Bio X Cell, BP0055) to differentiate CD4^+^ T cells into T_H_2 cells. Cells were supplemented with TGFβ (5 ng ml^−1^; Thermo Fisher Scientific, 100-21-100UG), IL-6 (10 ng ml^−1^; PeproTech, 216-16-100), IL-1β (10 μg ml^−1^; PeproTech, 100947), anti-IFNγ (10 μg ml^−1^; Bio X Cell, BP0055) and anti-IL-4 (10 μg ml^−1^; Bio X Cell, BP0045) to differentiate CD4^+^ T cells into T_H_17 cells. Cells were cultured under 5% CO_2_, atmospheric oxygen, at 37 °C in a humidified incubator for 4 days following activation. Where indicated cells were treated with vehicle control (DMSO), 1 μM (24 h) or 4 μM (6 h) alexidine dihydrochloride (Sigma, A8986), 1 µM ISRIB (Merk, SML0843), 1 μM oligomycin (Merck, O4876), 100 nM rotenone (Merck, R8875), 1 μM antimycin A (Merck, A8674), 50 μg ml^−1^ cycloheximide (Merck, C4859), 0.1 mM L-HPG (Sigma, 900893) and 50 μg ml^−1^ puromycin (Sigma, P9620).

#### CRISPR–CAS KO generation

The following gRNA was used to target the gene *Pgs1*: Mm.Cas9.PGS1.1.AA. The following guide was used as non-targeting control: Mm.Cas9.GCGAGGTATTCGGCTCCGCG. All guides were purchased from IDT. To prepare the targeting gRNA–Cas9 complex, equimolar amounts (180 pmol) of Alt-R CRISPR–Cas9 tracrRNA (IDT) and gene-specific crRNA (indicated above) were mixed, incubated for 5 min at 98 °C and cooled at room temperature for 20 min. In total, 60 pmol of recombinant Cas9 (IDT) was mixed and incubated for another 20 min. Naive CD4^+^ T cells (5 × 10^6^) isolated from PTPMT1 WT and PTPMT1 ΔT mice were electroporated using the P4 Primary Cell 4D-Nucleofector X Kit and the prepared gRNA–Cas9 complexes.

#### Suppression assay

After spleen processing, 50 × 10^3^ cells per well in triplicate were irradiated at 35 Gray and were used as feeder in culture. CD4^+^CD25^+^ T_reg_ cells (Stem Cell Technologies, 18783A) and CD8^+^ (target population; Stem Cell Technologies, 19853) were isolated from the total splenocytes with the corresponding T Cell Isolation Kit according to the manufacturer’s instructions. After cell count, to monitor the proliferation, CD8^+^ cells were incubated with 10 mM CFSE dye (Thermo Fisher Scientific, C34554) in complete RPMI medium, for 15 min at 37 °C (up to 15 × 10^6^ cells in 1 ml of medium), whereas T_reg_ cells were labelled in the same conditions with 10 mM Cell Trace Violet dye (Thermo Fisher Scientific, C34557). Following the incubation, an isovolume of FBS was added to both cell suspensions to stop the labelling, after centrifugation at 450*g* for 5 min, cells were washed in PBS 1× by centrifugation in 1600 RPMI for 10 min. Finally, CD8^+^ cells, T_reg_ cells and feeders were resuspended at a concentration of 50 × 10^3^ cells in 50 ml. T_reg_ cells and CD8^+^ cells were cultured at a constant 1:1 ratio with aCD3 (1 mg ml^−1^; Bio X Cell, BE0002). Cells were cultured for 4 days at 37 °C in a humidified 5% CO_2_ atmosphere. After culture, a multiparametric flow cytometry analysis was performed. The magnitude of proliferation was analysed by the dilution of the proliferation dyes in the gated T_reg_/CD8^+^ population.

#### Proliferation assay

Freshly isolated CD4^+^ T cells were stained with Cell Trace Violet (Thermo Fisher, C34557) according to manufacturer’s protocol. Briefly, cells were stained for 20 min in PBS with Cell Trace Violet. The reaction was stopped by adding a volume of RPMI + 10% FBS. Cells were then activated according to the CD4^+^ T cell activation and differentiation protocol. Four days after activation proliferation was measured as dye dilution by flow cytometry.

#### Flow cytometry

Fluorochrome-conjugated antibodies were purchased from eBioscience, BD Bioscience and BioLegend. Cells were detected using Fortessa flow cytometers and analysed using FlowJo software (BD Bioscience). Cell viability was quantified by flow cytometry using LIVE/DEAD aqua or near-IR dyes (Invitrogen) following the manufacturer’s instructions. For intracellular cytokine staining, cells were reactivated with 50 ng ml^−1^ phorbol 12-myristate 13-acetate + 500 ng ml^−1^ ionomycin (all Sigma) and cultured in the presence of Brefeldin A for 4 h before fixation using Cytofix Cytoperm (BD Bioscience).

#### Metabolic phenotyping

OCRs and ECARs were measured in XF media (non-buffered RPMI 1640 containing 25 mM glucose, 2 mM L-glutamine and 1 mM sodium pyruvate) under basal conditions and in response to 1 μM oligomycin, 1.5 μM FCCP and 100 nM rotenone + 1 μM antimycin A, using a 96-well XF or XFe Extracellular Flux Analyzer (EFA; Seahorse Bioscience). Around 2 × 10^5^ T cells per well were spun onto poly-D-lysine-coated Seahorse 96-well plates and preincubated at 37 °C for a minimum of 45 min in the absence of CO_2_.

#### Western blot

Cells were washed with ice-cold PBS 1× and lysed in RIPA lysis buffer 20 mM Tris-HCL (pH 7.5), 150 mM NaCl, 1 mM Na_2_EDTA, 1 mM EGTA, 1% Triton X-100, 2.5 mM sodium pyrophosphate, 1 mM β-glycerophosphate, 1 mM Na_3_VO_4_, 1 μg ml^−1^ leupeptin), supplemented with 1× Protease Inhibitor Cocktail (Cell Signaling, 5871) for 30 min, on ice, followed by centrifugation at 12,000*g* for 10 min, at 4 °C. Cleared protein lysate was quantified using Pierce BCA protein assay kit (23225) according to the manufacturer’s instruction. Cleared protein extracts were denatured with NuPAGE LDS loading buffer (Bio-Rad, 1610747) added with 50 mM DTT (Sigma-Aldrich, 10197777001) for 10 min at 75 °C and loaded onto precast NuPAGE 4–12% Bis-Tris protein gels (Thermo Fisher Scientific, NW04122BOX). Protein samples were run using MES buffer 1× (Thermo Fisher Scientific, NP0002) or MOPS buffer 1× (Life Technologies, NP0001). Proteins were transferred onto nitrocellulose membranes using the wet transfer in Transfer buffer (20% methanol, 1× Tris-Glycine SDS). Membranes were blocked for 1 h with 5% wt/vol BSA in TBS 1× Tween-20 0.05% and incubated with primary antibodies in 5% wt/vol BSA in TBS 1× Tween-20 0.05% overnight at 4 °C or 1 h at room temperature. All primary antibody incubations were followed by incubation with secondary horseradish peroxidase-conjugated antibodies (Pierce) in 5% wt/vol BSA in TBS 1× Tween-20 0.05% for 1 h at room temperature and visualized using SuperSignal West Pico or Femto Chemiluminescent Substrate (Pierce, 34580) using Imaging System Vilber Fusion Solos. The optical density of the signals on the film was quantified using grayscale measurements in ImageJ software (National Institutes of Health or NIH) and converted to fold change, normalized to the loading control.

#### Translation assays

To measure total translation, cells were treated with puromycin (10 µg ml^−1^) in complete culture medium for 30 min. Cells were washed with PBS and lysed, and protein concentration was determined. Equal amounts of protein (15 µg per lane) were resolved by SDS–PAGE and transferred to membranes. Membranes were incubated with anti-puromycin primary antibody (clone 12D10, Millipore, MABE343) followed by incubation with anti-mouse secondary antibody. visualized using SuperSignal West Pico Chemiluminescent Substrate (Pierce, 34580). To measure mitochondrial translation, after 1 h methionine starvation cells were pretreated 15 min with cycloheximide (CHX, Sigma, C4859) to inhibit cytoplasmic translation, then incubated for 2 h with the methionine alkyne analog L-HPG (Sigma, 900893) in presence of CHX. HPG incorporation was detected using a click chemistry approach conjugating the alkyne HPG with an Azide AF488 followed by FACS analysis.

#### RT–PCR

RNA was isolated using a RNeasy Mini Kit (74106, Qiagen) according to manufacturer’s instructions. RNA concentration was measured using Nanodrop 2000 (Thermo Fisher). cDNA synthesis was performed using High-Capacity cDNA Reverse Transcription Kit (4368813, Thermo Fisher) and RNasin Plus RNase Inhibitor (N2615, Promega), according to manufacturer’s instructions. Per reaction, 450–1000 ng RNA was used. RT-qPCR was performed in 384-wells plates using iTaqMan Universal Probes SuperMix (1725134, Bio-Rad), 10 ng cDNA per sample and 20× TaqMan Gene Expression Assay (Hprt: Mm03024075_m1, 4331182; Ddit3 (CHOP): Mm01135937_g, 4331182; Atf4: Mm00515325_g1, 4331182; Atf3: Mm00476033_m1, 4331182; Atf6: Mm01295319_m1, 4331182; Thermo Fisher). RT-qPCRs were performed with a QuantStudio 5 Real-Time PCR 384 well system (A28140, Thermo Fisher). Gene expression was normalized to Hprt expression. Fold change was calculated according to the delta-Ct method (2^−dCt^) or delta-delta-Ct method (2^−ddCt^).

#### Confocal and electron microscopy Imaging

For Confocal Microscopy imaging 3 × 10^6^ T_reg_ cells were collected after 4 days of culture and plated on Poly-D-lysine (50 mg ml^−1^; Thermo Fisher Scientific, A3890401) pre-coated slides (Lab-TekII, 154534). After fixation (20 min in formaldehyde 3.7%), cells were permeabilized using Nonidet 0.04% and then stained overnight at 4 °C with TOM20 antibody (Cruz Biotechnology, sc-11415). Samples were acquired using TCS SP8, Leica Microsystems (Confocal laser scanning microscope). Volume reconstructions and analysis were performed with Imaris software.

For Electron Microscope imaging 2 × 10^6^ T_reg_ cells were fixed 20 min RT in 2.5% glutaraldehyde in 100 mM sodium cacodylate, then washed in cacodylate buffer. After dehydration, samples were embedded in Eponate 12 resin (Ted Pella) and sections were cut. Images were acquired using a FEI Tecnai 12 Transmission electron microscope equipped with a TIETZ digital camera. Cristae width was measured using ImageJ software and averaged over 50 independent images, acquisition of EM micrographs and measurements of max cristae width displayed were performed using ImageJ software (NIH). Brightness and contrast were adjusted in ImageJ.

#### Tissue preparation for histology

Small intestines were dissected and washed with PBS. Small pieces (about 0.5 cm) of the small intestine were isolated from the proximal (after the stomach) and distal (before the caecum) regions, and the tissue was cut longitudinally and washed in PBS to remove faeces. Intestinal tissue samples were rolled up from proximal to distal to form a Swiss roll and either fixed in 4% paraformaldehyde overnight at 4 °C or embedded in Tissue-Tek for cryo sectioning.

#### H&E staining of paraffin-fixed tissues

Paraffin-embedded 3-μm-thick intestinal tissue sections were deparaffinized with xylene and rehydrated with decreasing amounts of ethanol solutions (100% ethanol, 96% ethanol and 75% ethanol). Sections were stained for 2 min in haematoxylin, differentiated in tap water for 15 min and incubated for 1 min in eosin. Stained sections were dehydrated using increasing amounts of ethanol solutions and fixed in xylene for 1 min. Slides were mounted with Entellan.

#### PAS staining on paraffin-fixed tissues

Paraffin-embedded tissue sections were deparaffinized and rehydrated to distilled water. Sections were incubated with freshly prepared periodic acid solution for 5–8 min, followed by two washes in distilled water (5 min each). Slides were then incubated with Schiff’s reagent for up to 20 min and washed under running tap water for 10 min. Nuclei were counterstained with haematoxylin for 5 min and rinsed under running tap water for an additional 10 min. Sections were dehydrated through graded ethanol, cleared in xylol and mounted using Eukitt or Entellan.

#### Immunohistochemistry on intestinal sections

Paraffin sections were rehydrated, and heat-induced antigen retrieval was performed in 10 mM sodium citrate, 0.05% Tween-20 at pH 6.2 or with proteinase K treatment. Endogenous peroxidase was blocked in peroxidase blocking buffer for 15 min at room temperature. Sections were blocked in 1% BSA, 0.2% fish-skin gelatine, 0.2% Triton X-100 and 0.05% Tween-20 in PBS for 1 h at room temperature. After blocking, the sections were incubated overnight at 4 °C with primary antibodies against CD45 (BD Bioscience, 560510, clone 30-F11, 1:500 dilution), F4/80 (AbD Serotec, MCA497, clone A3-1, 1:1,000 dilution) and CD3 (Ab5690). Sections were incubated with biotinylated anti-mouse IgG (H + L; Vector Laboratories, BA-9200-1.5, 1:1,000 dilution), anti-rabbit IgG (H + L; Vector Laboratories, BA-1000-1.5, 1:1,000 dilution) and anti-rat IgG (H + L; Vector Laboratories, BA-9400-1.5, 1:1,000 dilution) secondary antibodies. Each staining was visualized using ABC Kit Vectastain Elite (Vector, PK6100) and DAB substrate (Dako and Vector Laboratories). For image acquisition, the intestinal sections were analysed using a light microscope equipped with a KY-F75U digital camera (JVC; DM4000B, Leica Microsystems, Diskus 4.50 software), a TCS SP8 confocal laser scanning microscope (Inverse, DMi 8 CS, Leica Microsystems LAS X, Lightning software v.5.1.0). Each data point corresponds to the average values from at least three randomly selected intestinal areas of a single mouse. Representative pictures from three mice per genotype per time point were analysed.

#### Histopathological scoring

Histopathological scoring was performed using a composite system assessing epithelial hyperplasia, goblet cell depletion, inflammation and tissue involvement. Epithelial hyperplasia was scored from 0 to 3 (0, none; 1, mild, ~1.5×; 2, moderate, 2–3×; 3, severe, >3×). Goblet cell depletion was scored from 0 to 3 based on the percentage of loss (0, none; 1, mild, ~25%; 2, marked, 25–50%; 3, substantial, >50%). Inflammation in the lamina propria was graded from 0 to 3 (0, none or few leucocytes; 1, mild increase in leucocytes at crypt tips or presence of multiple lymphoid follicles; 2, moderate, marked infiltrate with notable crypt broadening; 3, severe, dense infiltrate throughout). The area affected was scored according to the percentage of the section involved.

#### Lipidomics

Mass spectrometry (MS)-based lipid analysis was performed by Lipotype as described^75^. Lipids from 10 × 10^6^ CD4^+^ T_reg_ cells per sample were extracted using a chloroform–methanol procedure^76^. Samples were spiked with internal lipid standard mixture containing: cardiolipin 14:0/14:0/14:0/14:0 (CL), ceramide 18:1;2/17:0 (Cer), diacylglycerol 17:0/17:0 (DAG), hexosylceramide 18:1;2/12:0 (HexCer), lyso-phosphatidate 17:0 (LPA), lyso-phosphatidylcholine 12:0 (LPC), lyso-phosphatidylethanolamine 17:1 (LPE), lyso-phosphatidylglycerol 17:1 (LPG), lyso-phosphatidylinositol 17:1 (LPI), lyso-phosphatidylserine 17:1 (LPS), phosphatidate 17:0/17:0 (PA), phosphatidylcholine 15:0/18:1 D7 (PC), phosphatidylethanolamine 17:0/17:0 (PE), phosphatidylglycerol 17:0/17:0 (PG), phosphatidylinositol 16:0/16:0 (PI), phosphatidylserine 17:0/17:0 (PS), cholesterol ester 16:0 D7 (CE), sphingomyelin 18:1;2/12:0;0 (SM) and triacylglycerol 17:0/17:0/17:0 (TAG). After extraction, the organic phase was transferred to an infusion plate and dried in a speed vacuum concentrator. The dry extract was resuspended in 7.5 mM ammonium formate in chloroform–methanol–propanol (1:2:4; vol:vol:vol). All liquid handling steps were performed using Hamilton Robotics STARlet robotic platform with the Anti Droplet Control feature for organic solvents pipetting. Samples were analysed by direct infusion on a QExactive mass spectrometer (Thermo Scientific) equipped with a TriVersa NanoMate ion source (Advion Biosciences). Samples were analysed in both positive and negative ion modes with a resolution of R*m/z* = 200 = 280,000 for MS and R*m/z* = 200 = 17,500 for MS/MS experiments, in a single acquisition. MS/MS was triggered by an inclusion list encompassing corresponding MS mass ranges scanned in 1-Da increments^77^. Both MS and MS/MS data were combined to monitor CE, DAG, TAG and TAG O- ions as ammonium adducts; LPC, LPC O-, PC and PC O- as formiate adducts; and CL, LPS, PA, PE, PE O-, PG, PI and PS as deprotonated anions. MS only was used to monitor LPA, LPE, LPE O-, LPG and LPI as deprotonated anions, and Cer, HexCer and SM as formiate adducts. Data were analysed using LipotypeXplorer, a proprietary software developed by Lipotype, which is based on LipidXplorer^78,79^. Data post-processing and normalization were performed using an in-house developed data management system. Only lipid identifications with a signal-to-noise ratio > 5 and a signal intensity fivefold higher than in corresponding blank samples were considered for further data analysis. PGPs were identified based on the accurate *m/z* of the precursor ion, species-specific fatty acids and class-specific head group fragments. Quantification was performed based on the signals of the precursor ions of the reported species.

#### Proteomics

CD4^+^ T_reg_ cells (4 × 10^6^) were lysed in 8 M urea/50 mM TEAB buffer and sonified using Bioruptor (10 min, cycle 30/30 s). After centrifugation at 20,000*g* for 15 min, proteins were quantified. DTT to a final concentration of 5 mM was added to 50 µg of proteins and incubated at 25 °C for 1 h. CAA was then added to a final concentration of 40 mM, vortexed and incubated in the dark for 30 min. Lys-C protease was then added at an enzyme:substrate ratio of 1:75 and incubated at 25 °C for 4 h. After 4 h samples were diluted with 50 mM TEAB buffer to achieve a final concentration of urea ≤ 2 M. Trypsin was finally added at an enzyme:substrate ratio of 1:75 and incubated at 25 °C overnight. Then, formic acid was added to the samples to a final concentration of 1% and samples were loaded on SDB-RP stage tips. Samples were analysed by the CECAD Proteomics Facility on an Orbitrap Exploris 480 (Thermo Scientific) mass spectrometer equipped with a FAIMS pro differential ion mobility device that was coupled to an UltiMate 3000 (Thermo Scientific). Samples were loaded onto a precolumn (Acclaim 5 µm PepMap 300 µm Cartridge) for 2 min at 15 µl flow before being reverse-flushed onto an in-house packed analytical column (30 cm in length, 75-µm inner diameter, filled with 2.7 µm Poroshell EC120 C18, Agilent). Peptides were chromatographically separated at a constant flow rate of 300 nl min^−1^ and the following gradient: initial 6% B (0.1% formic acid in 80% acetonitrile), up to 32% B in 72 min, up to 55% B within 7 min and up to 95% solvent B within 2 min, followed by column wash with 95% solvent B and re-equilibration to initial condition. The FAIMS pro was operated at −50 V compensation voltage and electrode temperatures of 99.5 °C for the inner and 85 °C for the outer electrode. MS1 scans were acquired from 399 *m/z* to 1,001 *m/z* at at a resolution of 15,000. Maximum injection time was set to 22 ms and the automatic gain control target to 100%. MS2 scans ranged from 400 *m/z* to 1,000 *m/z* and were acquired at a resolution of 15,000 with a maximum injection time of 22 ms and an automatic gain control target of 100%. DIA scans covering the precursor range of 400–1,000 *m/z* were acquired in 60 × 10-*m/z* windows with an overlap of 1 *m/z*. All scans were stored as centroid. Samples were analysed in DIA-NN (1.8.1)^80^. A Swissprot mouse canonical database (UP589, downloaded 18 June 2020) was used for library building with settings matching acquisition parameters and the match-between-runs function enabled. Here, samples are directly used to refine the library for a second search of the sample data. DIA-NN was run with the additional command line prompts ‘--report-lib-info’ and ‘--relaxed-prot-inf’. Further output settings were: filtered at 0.01 FDR; N-terminal methionine excision enabled; maximum number of missed cleavages set to 1; minimum peptide length set to 7; maximum peptide length set to 30; minimum precursor *m/z* set to 400; maximum precursor *m/z* set to 1,000; cysteine carbamidomethylation enabled as a fixed modification. Afterwards, DIA-NN output was further filtered on library *q* value and global *q* value ≤ 0.01 and at least two unique peptides per protein using R (4.1.3). Finally, LFQ values were calculated using the DIA-NN R package. Afterwards, analysis of results was performed in Perseus (1.6.15)^81^. FDR *q* values in gene-set enrichment analysis were calculated as previously described^82^. Proteomics datasets are available in PRIDE under accession numbers PXD060518 and PXD071483.

#### RNA-seq and cluster analysis

Libraries were prepared using the Illumina Stranded TruSeq RNA sample preparation Kit. Library preparation started with 500 ng total RNA. After poly-A selection (poly-T oligo-attached magnetic beads), mRNA was purified and fragmented using divalent cations under elevated temperature. The RNA fragments underwent reverse transcription using random primers. This was followed by second-strand cDNA synthesis with DNA Polymerase I and RNase H. After end repair and A-tailing, indexing adaptors were ligated. The products were then purified and amplified (15 PCR cycles) to create the final cDNA libraries. After library validation and quantification (Agilent Tape Station), equimolar amounts of the library were pooled. The pools were quantified using the Peqlab KAPA Library Quantification Kit and the Applied Biosystems 7900HT Sequence Detection System. The pool was sequenced on an Illumina NovaSeq 6000 sequencing instrument with a PE100 protocol. RNA-seq data were analysed using the Cologne Center for Genomics’ in-house pipeline for RNA-seq analysis based on the Nextflow DSL (version 20.01.0 build 5264). In short, fastq files were adaptor-trimmed with trimmomatic (v0.38), removing sequences of length < 18 nucleotides after trimming, then mapped against the hg38 human reference genome and the gene assembly v101 using STAR aligner (v2.6.1a) with the default settings. The count matrix was generated using subread (v1.6.4) and DEGs were called with DeSeq2 (v1.20.0) with an adjusted *P*-value cut-off of 0.05. Overrepresented Gene Ontology biological processes and KEGG pathways were discovered using clusterprofiler (v3.17.0) on upregulated or downregulated DEGs (with an adjusted *P* value < 0.05 and a fold change of >2 for either upregulated or downregulated). Volcano plots were generated with bioconductor package enchancedVolcano (v1.4.0; https://github.com/kevinblighe/EnhancedVolcano) and heat maps with the heatmap.2 or pheatmap sub-function of gplots (v3.1.0 or v1.0.12, respectively; https://github.com/talgalili/gplots/ and https://github.com/raivokolde/pheatmap/, respectively). For the analysis of the transcriptomic profile of WT, CHOP KO, PTPMT1 ΔT and DKO T_reg_ cells, the raw fastq files were processed with the nf-core/RNA-seq pipeline under Nextflow v25.04.6 build 5954 on the ITCC HPC cluster. The counts were processed using a custom DESeq2 pipeline. In short, we processed the generated gene counts table (rsem.merged.gene_counts.tsv) from the nf-core/RNA-seq pipeline with DESeq2. We extracted the genes that were significantly differentially expressed in the pairwise comparison of each condition (1,148 genes that survived the adjusted *P* value < 0.05 and |logFC | >1 thresholds) and performed cluster analysis using the degPatterns function from the DEGreport package (v1.42), using the following parameters: summarize = ‘merge’, minc = 15, time = ‘group’, col = ‘group’, scale = ,plot = TRUE. For the ORA analysis of the genes from the annotated clusters, we used a custom function based on the enricher function from the clusterProfiler package (v4.14.6). For the background (universe) genes, we used the genes with no *P* value, as identified by the independent filtering method included in the DESeq2 pipeline. The gene sets were extracted using the msigdbr function (v.25.1.1). FDR *q* values in gene-set enrichment analysis were calculated as previously described^82^. RNA-seq datasets are available in the Gene Expression Omnibus (GEO) under accession numbers GSE288709 and GSE314259.

#### Cytokine measurements

Cytokine concentration was measured from mouse using Luminex xMAP technology for multiplexed quantification of 32 mouse cytokines, chemokines and growth factors. The multiplexing analysis was performed using the Luminex 200 system (Luminex) by Eve Technologies. Thirty-two markers were simultaneously measured in the samples using Eve Technologies’ Mouse Cytokine 32-Plex Discovery Assay (MilliporeSigma) according to the manufacturer’s protocol.

#### Metagenomics

Raw reads from Illumina were demultiplexed and their quality evaluated with FastQC (v0.12)^83^ and multiqc (v1.14)^84^. Sequence adaptors were trimmed and filtered following standard procedures by using Trimmomatic (v0.39)^85^. Sequences coming from the host were mapped against the *Mus musculus* reference genome (GRCm38) and dropped using bowtie2 (v.2.5.4)^86^. After pre-processing the reads, taxonomy assignment was done with MetaPhlAn 4.06 using default parameters^87^ to capture the species level with high quality. Taxonomy and OTU table were then processed and analysed in R. The main analysis was done using tidyverse v.2.0.1 and microeco (v.1.6.0)^88^. To capture the beta diversity of the samples, Bray–Curtis distances were used to calculate the principal coordinate analysis. Differences between the relative abundance of species were calculated using the Wilcoxon statistical test. Graphical representation of the results was done with the R package ggplot2 v.3.5.1.

#### scRNA-seq

CD45^+^ single cells were sorted with BD FACSAria Cell Sorter and prepared for scRNA-seq using the 10x Genomics 3′ scRNA v3 system. Libraries were sequenced on a NovaSeq 6000 at 250 million reads per sample. Samples were demultiplexed, quality checked, filtered and aligned to the genome build GRCm38 using pre-established pipelines implemented via Cell Ranger v.8.0.1. The resulting raw read count matrix of barcodes corresponding to cells and features corresponding to detected genes were processed, analysed and visualized in R v.4.3.1 using Seurat (v.5)^89^ with default parameters in all functions, unless specified. Poor-quality cells, with low total unique molecular identifier counts and high percentage mitochondrial gene expression, were excluded. Filtered samples were normalized using a regularized negative binomial regression (SCTransform)^90^ and integrated with Harmony^91^, using 50 principal components. Integrated gene expression matrices were visualized with a UMAP^92^ as a dimensionality reduction approach. Resolution for cell clustering was determined by evaluating hierarchical clustering trees at a range of resolutions (0–1.2) with Clustree^93^ and by calculating a silhouette score, selecting a value inducing minimal cluster instability and maintaining a high proportion of cells with a positive silhouette score. DEGs between clusters were identified as those expressed in at least 10% of cells with a greater than +1 log fold change and an adjusted *P* value of less than 0.01, by transforming read count matrices to pseudobulk using Libra (https://github.com/neurorestore/Libra/) and then applying DESeq2 (ref. ^94^) v.1.36 with default parameters. Artificial replicates for differential gene expression analysis were generated by randomly partitioning the single-cell dataset into three tiles. Ribosomal protein genes were excluded from results. Cluster-specific genes were explored for pathway enrichment using StringDB^95^. Gene-set scores were calculated using the UCell with default parameters^96^. The scRNA-seq dataset is available with accession number GSE288641.

#### Blue Native PAGE and immunoblotting assay

About 30 × 10^6^ T cells were permeabilized with 200 μl of PBS and 200 μl of 8 mg ml^−1^ digitonin at 4 °C for 10 min. A total of 1 ml of PBS was added to block the digitonization, and samples were centrifuged for 10 min at 10,000*g*. Pellets were washed with 1 ml PBS, and after centrifugation, were resuspended in 80 μl of 1.5 M of aminocaproic acid, 50 mM Bis-Tris/HCl (pH 7) with 10 μl of digitonin at 10% (in aminocaproic acid). After incubation for 5 min at 4 °C, samples were centrifuged for 20 min at maximum speed. The supernatant was collected and mixed with 10 μl of 5% Serva Blue G dye (in 1 M 6-aminohexanoic acid). In total, 25 µl of the mitochondrial sample was separated by 3–13% gradient Blue Native gel^97^. After electrophoresis, gels were electroblotted onto Hybond-P-polyvinylidene fluoride (PVDF) membranes (GE Healthcare) and immunoblotted with specific antibodies for the different complexes of mitochondrial respiratory chain: anti-NDUFA9, for complex I (Abcam, ab14713); anti-MTCO1 (1D6E1A8) for detection of complex IV (Invitrogen, 459600); anti-UQCRC2, for detection of complex III (Proteintech, 14742-1-AP); anti-SDHA for complex II (Invitrogen 459200); and anti-VDAC1 (Abcam, ab15895) for normalization. Fluorescence secondary antibodies were anti-rabbit IgG (H + L) cross-adsorbed secondary antibody, Alexa Fluor 680 (A21076, Invitrogen), and anti-mouse IgG (H + L) cross-adsorbed secondary antibody, DyLight 800 (SA5-10176, Invitrogen). Signals were detected and quantified using Odyssey CLX (LI-COR).

#### Clinical information of patients with Barth syndrome

The Barth Syndrome Registry, organized and managed by the Barth Syndrome Foundation (https://www.barthsyndrome.org/), collects information on patients with a confirmed genetic diagnosis of Barth syndrome. The Barth Syndrome Registry was approved by the Institutional Review Boards of the two participating institutions: University of Florida and Boston Children’s Hospital. Inclusion criteria for the Registry are a diagnosis of Barth syndrome, *TAZ* gene mutation and the provision of informed consent. Data from the Barth Syndrome Registry presented herein are either extracted in an anonymized fashion from the registry or from the Barth Syndrome Registry reports^58,59^.

#### Quantification and statistical analysis

Flow cytometry data were analysed using FlowJo 10 (BD Biosciences). Statistical analyses were performed using Prism 7 software (GraphPad) and results are represented as the mean ± s.e.m., unless otherwise indicated. Comparisons for two groups were calculated using unpaired two-tailed Student’s *t*-tests. Comparisons of more than two groups were calculated using one-way ANOVA with Tukey’s or Dunn’s multiple-comparison tests. We observed normal distribution and no difference in variance between groups in individual comparisons. Selection of sample size was based on extensive experience with metabolic assays. The sample size for in vivo experiments was based on previous experience with infection experiments. The number of independent experiments performed, and the *P* values for each experiment are reported in the corresponding figure legends. For both in vitro and in vivo experiments, no initial exclusion criteria were used, and no animals or replicates were excluded from the study.

#### Data collection and randomization

Mice or cell culture samples or dishes were randomly assigned to experimental groups. Data collection and outcome assessments were performed in a randomized manner, when applicable. Experimental conditions and sample processing were organized to minimize bias, with randomization applied during sample allocation and data acquisition. The data distribution was assumed to be normal, but this was not formally tested. Researchers performing data collection and analysis were not blinded to experimental conditions.

### Reporting summary

Further information on research design is available in the Nature Portfolio Reporting Summary linked to this article.

## Supplementary information

Supplementary Information Supplementary Fig. 1.

Reporting Summary

## Source data

Source Data Fig. 1 Statistical source data and tissue images.

Source Data Fig. 2 Statistical source data and tissue images.

Source Data Fig. 3 Statistical source data and tissue images.

Source Data Fig. 4 Statistical source data.

Source Data Fig. 5 Statistical source data and uncropped blot scans.

Source Data Fig. 6 Statistical source data and tissue images.

Source Data Fig. 7 Statistical source data.

Source Data Extended Data Fig. 1 Statistical source data.

Source Data Extended Data Fig. 2 Statistical source data.

Source Data Extended Data Fig. 3 Statistical source data.

Source Data Extended Data Fig. 4 Statistical source data.

Source Data Extended Data Fig. 5 Statistical source data.

Source Data Extended Data Fig. 6 Statistical source data and uncropped blot scans.

Source Data Extended Data Fig. 7 Statistical source data.

Source Data Figure 5, Extended Data 5, Extended Data 6 Uncropped blots.

## Data Availability

RNA-seq datasets are available under GEO accession numbers GSE288709 and GSE314259. The scRNA-seq dataset is available with accession number GSE288641. Proteomics datasets are available in PRIDE under accession numbers PXD060518 and PXD071483. Source data are provided with this paper.

## Code availability

All analyses were performed using standard software packages as described in the Methods.
