# Supplementary material for: Cardiolipin preserves Treg metabolic fitness and immune homeostasis in the gut
Source: Nat Metab. 2026 May 18;8(6):1368–89. doi: 10.1038/s42255-026-01533-9 (PMC13303090; doi:10.1038/s42255-026-01533-9)
Supplement: Supplementary file 1 — Supplementary Fig. 1. [file 42255_2026_1533_MOESM1_ESM.pdf]

nature metabolism

Supplementary information https://doi.org/10.1038/s42255-026-01533-9

Cardiolipin preserves T_{reg} metabolic fitness and immune homeostasis in the gut

In the format provided by the authors and unedited

Fig Supplementary 1

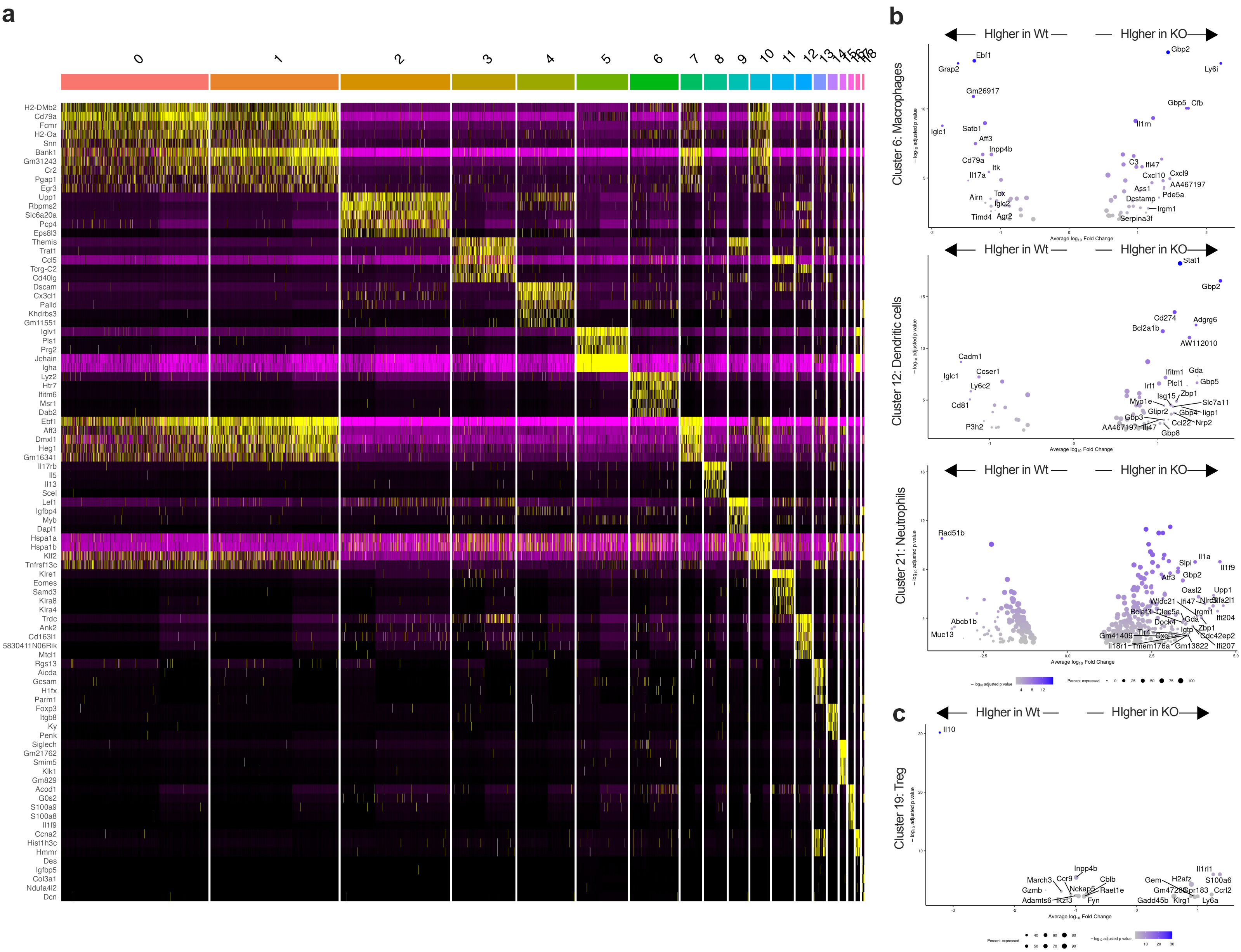

Figure Supplementary 1

(**a**) Cluster analysis depicting the first 5 most differently expressed genes (DEGs) per cluster of CD45^{+}-sorted cells from PTPMT1 Wt and PTPMT1 ΔT siLP. (**b**) Volcano plots depicting DEGs between PTPMT1 Wt and PTPMT1 ΔT macrophages, dendritic cells and neutrophils in the scRNAseq dataset. (**c**) Volcano plots depicting DEGs between PTPMT1 Wt and PTPMT1 ΔT Treg cells in the scRNAseq dataset.
