# Supplementary material for: Cardiolipin preserves Treg metabolic fitness and immune homeostasis in the gut
Source: Nat Metab. 2026 May 18;8(6):1368–89. doi: 10.1038/s42255-026-01533-9 (PMC13303090; doi:10.1038/s42255-026-01533-9)
Supplement: Supplementary file 2 — Reporting Summary [file 42255_2026_1533_MOESM2_ESM.pdf]

nature portfolio

Corresponding author(s): CORRADO Mauro

Last updated by author(s): Mar 22, 2026

Reporting Summary

Nature Portfolio wishes to improve the reproducibility of the work that we publish. This form provides structure for consistency and transparency in reporting. For further information on Nature Portfolio policies, see our Editorial Policies and the Editorial Policy Checklist.

Statistics

For all statistical analyses, confirm that the following items are present in the figure legend, table legend, main text, or Methods section.

n/a	Confirmed	
☐	☒	The exact sample size (n) for each experimental group/condition, given as a discrete number and unit of measurement
☐	☒	A statement on whether measurements were taken from distinct samples or whether the same sample was measured repeatedly
☐	☒	The statistical test(s) used AND whether they are one- or two-sided<br>*Only common tests should be described solely by name; describe more complex techniques in the Methods section.*
☒	☐	A description of all covariates tested
☐	☒	A description of any assumptions or corrections, such as tests of normality and adjustment for multiple comparisons
☐	☒	A full description of the statistical parameters including central tendency (e.g. means) or other basic estimates (e.g. regression coefficient) AND variation (e.g. standard deviation) or associated estimates of uncertainty (e.g. confidence intervals)
☐	☒	For null hypothesis testing, the test statistic (e.g. F, t, r) with confidence intervals, effect sizes, degrees of freedom and P value noted<br>*Give P values as exact values whenever suitable.*
☒	☐	For Bayesian analysis, information on the choice of priors and Markov chain Monte Carlo settings
☒	☐	For hierarchical and complex designs, identification of the appropriate level for tests and full reporting of outcomes
☒	☐	Estimates of effect sizes (e.g. Cohen's d, Pearson's r), indicating how they were calculated

Our web collection on statistics for biologists contains articles on many of the points above.

Software and code

Policy information about availability of computer code

Data collection	LSR Fortessa flow cytometer, BD Biosciences<br>FACSAria III cell sorter, BD Biosciences<br>XFe96 extracellular flux analyzer, Agilent<br>Imaging System Vilber Fusion Solos<br>QuantStudioTM 5 Real-Time PCR 384 well system , Thermofisher<br>SP8, Leica Microsystems<br>FEI Tecnai 12 Transmission electron microscope equipped with a TIETZ digital camera<br>DM4000B, Leica Microsystems<br>Orbitrap Exploris 480, Thermo Scientific<br>Illumina NovaSeq6000<br>Luminex™ 200 System, Luminex<br>Odyssey CLX, LI-COR<br>Cytek Aurora
Data analysis	FlowJo<br>Fiji Imaging<br>GraphPad Prism<br>DIA-NN 1.8.1<br>R v4.3.1<br>Perseus 1.6.15<br>Trimmomatic (v0.38)

Trimmomatic (v0.39)
STAR aligner (v2.6.1a)
FastQC (v0.12)
DeSeq2 (v1.20.0)
Clusterprofiler (v3.17.0)
Multiqc (v1.14)
Bowtie2 (v.2.5.4)
MetaPhlAn 4.06
Cellranger (v. 8.0.1)
Seurat v.5
DESeq2 v_1.36
StringDB
Cytolution (Cytolytics)

For manuscripts utilizing custom algorithms or software that are central to the research but not yet described in published literature, software must be made available to editors and reviewers. We strongly encourage code deposition in a community repository (e.g. GitHub). See the Nature Portfolio guidelines for submitting code & software for further information.

Data

Policy information about availability of data

All manuscripts must include a data availability statement. This statement should provide the following information, where applicable:

- Accession codes, unique identifiers, or web links for publicly available datasets
- A description of any restrictions on data availability
- For clinical datasets or third party data, please ensure that the statement adheres to our policy

RNAseq datasets are available with GEO accession number GSE288709 and GSE314259. scRNAseq dataset is available with accession number GSE288641. Proteomics datasets are available in PRIDE with accession number PXD060518 and PXD071483. Source data are provided with this paper.

Research involving human participants, their data, or biological material

Policy information about studies with human participants or human data. See also policy information about sex, gender (identity/presentation), and sexual orientation and race, ethnicity and racism.

Reporting on sex and gender	The reported research on human samples is focused on an X-linked genetic disorder that only affects genetically male individuals, with extremely rare exceptions. PBMCs from healthy donors and Barth Syndrome patients were collected at University of Bristol in accordance with the Helsinki Declaration with approval from the UK NHS Research Ethics committee (permit number 09/H0202/52)
Reporting on race, ethnicity, or other socially relevant groupings	N/A
Population characteristics	N/A
Recruitment	N/A
Ethics oversight	UK NHS Research Ethics committee (permit number 09/H0202/52)

Note that full information on the approval of the study protocol must also be provided in the manuscript.

Field-specific reporting

Please select the one below that is the best fit for your research. If you are not sure, read the appropriate sections before making your selection.

☒ Life sciences ☐ Behavioural & social sciences ☐ Ecological, evolutionary & environmental sciences

For a reference copy of the document with all sections, see nature.com/documents/nr-reporting-summary-flat.pdf

Life sciences study design

All studies must disclose on these points even when the disclosure is negative.

Sample size	No sample-size calculations were performed, and sample sizes were arbitrarily chosen according to convention in the field. The number of replicates is at least 3 biological replicates and experiments were performed at least 3 times independently with at least 3 technical replicates (= repeated measurements of the same original sample), unless otherwise indicated in each figure legend.
Data exclusions	No data were excluded
Replication	All in vitro experiments were performed with a minimum of 3 independent biological replicates unless otherwise noted. All experiments were

Replication	repeated at least three times otherwise noted in figure legend.
Randomization	Randomization is not appropriate for this type of study. Samples were collected and analyzed with replicates for each studied genotype as described.
Blinding	Researchers performing data collection and analysis were not blinded to experimental conditions.

Reporting for specific materials, systems and methods

We require information from authors about some types of materials, experimental systems and methods used in many studies. Here, indicate whether each material, system or method listed is relevant to your study. If you are not sure if a list item applies to your research, read the appropriate section before selecting a response.

Materials & experimental systems

n/a	Involved in the study	
☐	☒	Antibodies
☐	☒	Eukaryotic cell lines
☒	☐	Palaeontology and archaeology
☐	☒	Animals and other organisms
☒	☐	Clinical data
☒	☐	Dual use research of concern
☒	☐	Plants

Methods

n/a	Involved in the study	
☒	☐	ChIP-seq
☐	☒	Flow cytometry
☒	☐	MRI-based neuroimaging

Antibodies

Antibodies used

Alexa Fluor® 488 anti-mouse CD4 Antibody, Biolegend, Cat#100423;
PE-Cy7 anti-mouse CD4, Biolegend, Cat#100528;
PE anti-mouse CD278 (ICOS), Biolegend, Cat#313508;
APC anti-mouse CD25, Biolegend, Cat#101910;
PE-Cy7 anti-mouse CD357, Invitrogen, Cat#25-5874-82
PerCP-Cy™5.5 Rat Anti-Mouse FoxP3, BD, Cat#563902;
Brilliant Violet 605™ anti-mouse CD152, Biolegend, Cat#106323;
PE/Cy7 anti-mouse CD8a, Biolegend, Cat#100722;
FITC anti-mouse CD45, Biolegend, Cat#103108;
PE anti-mouse CD45, Biolegend, Cat#103423;
PerCP/Cyanine5.5 anti-mouse CD8b, Biolegend, Cat#126610;
APC-Cy™7 Rat Anti-Mouse CD4, Biolegend, Cat#561830;
PE/Cyanine7 anti-mouse TCR β chain, Biolegend, Cat#109222;
PerCP/Cyanine5.5 anti-mouse TCR β chain, Biolegend, Cat#109228;
PE anti-mouse FOXP3, Biolegend, Cat#126404;
PE/Cyanine7 anti-T-bet, Biolegend, Cat#644824;
eFluor™ 660 Gata-3 Monoclonal, Biolegend, Cat#50-9966-42;
Alexa Fluor® 647 anti-mouse Ly-6G/Ly-6C (Gr-1), Biolegend, Cat#108420;
PE Rat Anti-Mouse CD19, BD, Cat#553786;
PerCP-Cy™5.5 Rat Anti-Mouse Siglec-F, BD, Cat#565526;
APC anti MHC Class II (I-A/I-E) Monoclonal Antibody (M5/114.15.2), Thermofisher, Cat#17-5321-82;
APC/Cyanine7 anti-mouse/human CD11b, Biolegend, Cat#101226;
PE anti-mouse Ly-6C, Biolegend, Cat#128007;
PE/Cyanine7 anti-mouse F4/80, Biolegend, Cat#123114;
Pacific Blue™ anti-mouse CD11c, Biolegend, Cat#117322;
PE anti-mouse CD62L, Biolegend, Cat#104408;
FITC anti-mouse/human CD44, Biolegend, Cat#103006;
FITC Anti-IFN-g Mouse Monoclonal Antibody, Biolegend, Cat#502507;
PE anti-mouse IL-17A, Biolegend, Cat#506904;
Alexa Fluor® 647 anti-mouse TNF-α, Biolegend, Cat#506314;

Validation

All antibodies were validated in cells not expressing the specific antibody target. These non expressing cells were also used to set negative control.

Eukaryotic cell lines

Policy information about cell lines and Sex and Gender in Research

Cell line source(s)	All in vitro experiments in the manuscript use primary mouse CD4 T cells isolated form mice of the indicated genotypes.
Authentication	Purity of primary CD4 T cell isolation was validated by FACS. KO were validated by qPCR and WB.

Mycoplasma contamination	All cell lines were routinely tested and confirmed negative for mycoplasma contamination by PCR.
Commonly misidentified lines (See ICLAC register)	N/A

Animals and other research organisms

Policy information about studies involving animals; ARRIVE guidelines recommended for reporting animal research, and Sex and Gender in Research

Laboratory animals	PTPMT1 floxed (RRID: IMSR_JAX:020775) and CHOP-/- (RRID: IMSR_JAX:005530) mice were purchased from The Jackson Laboratory. cGAS-/- were generated in house by ivRF (in vivo Research Facility) CECAD and kindly donated by Dr. Manolis Pasparakis a. PTPMT1 floxed mice were crossed to CD4-Cre mice as previously described (Corrado et al., Cell Metabolism, 2020) or to Foxp3-YFP-CRE (RRID:IMSR_JAX:016959) kindly donated by Dr. Marc Beyer (DZNE, Bonn, Germany). Age and sex of the mice is indicated either in figure legend or main text.
Wild animals	N/A
Reporting on sex	Lifespan studies and main immunophenotyping in PTPMT1 DT mice were performed in male and female mice and results are provided in main figures or supplementary ones. Analysis of PTPMT1 FoxP3-CRE and TAZ KO mice was performed only in male mice as only male mice develop a phenotype because X chromosome-linked. Analysis of other mouse strains focused only in males where the phenotype was more severe as stated in the manuscript.
Field-collected samples	N/A
Ethics oversight	Breeding of the animals was approved by Landesamt für Natur, Umwelt und Verbraucherschutz Nordrhein-Westfalen (LANUV NRW). EAE, L.monocytogenes and CD4 T cells-mediated colitis experimental studies were approved by the Regierungspräsidium Freiburg. H.hepaticus infection was conducted at University of Oxford in accordance with the UK Scientific Procedures Act of 1986, and by persons holding a personal license. The project license governing the mouse studies was reviewed by the University of Oxford's Animal Welfare and Ethical Review Board and approved by the Home Office of His Majesty's Government.

Note that full information on the approval of the study protocol must also be provided in the manuscript.

Plants

Seed stocks	N/A
Novel plant genotypes	N/A
Authentication	N/A

Flow Cytometry

Plots

Confirm that:

- [ ] The axis labels state the marker and fluorochrome used (e.g. CD4-FITC).
- [x] The axis scales are clearly visible. Include numbers along axes only for bottom left plot of group (a 'group' is an analysis of identical markers).
- [x] All plots are contour plots with outliers or pseudocolor plots.
- [x] A numerical value for number of cells or percentage (with statistics) is provided.

Methodology

Sample preparation	Cell viability was quantified by flow cytometry using LIVE/DEADTM aqua or near-IR dyes (Invitrogen) following manufacturer instructions. For intracellular cytokine staining cells were reactivated with 50 ng/mL phorbol 12-myristate 13-acetate (PMA) + 500 ng/mL ionomycin (all Sigma) and cultured in the presence of Brefeldin A for 4 h prior to fixation. For surface marker, cells were incubated first with LIVE Dead staining in PBS, then with surface marker antibody cocktail in PBS + 1mM EDTA + 2%FCS.
Instrument	LSR Fortessa flow cytometer, BD Biosciences<br>FACSAria III cell sorter, BD Biosciences<br>Cytek Aurora

Software	Data were acquired using FACSDIVA software with automated compensation (performed independently for each experiment with single-stained samples following the software assistant) and analyzed using FlowJo software
Cell population abundance	In in vitro experiments a stopping recording target was set at 20.000 events in target gate. For ex vivo analysis from lamina propria or other organs, all samples were acquired with a minimum of 500.000 events acquired.
Gating strategy	Cell discrimination was done applying the following gating strategy: 1) lymphoid cells were identified based on forward scatter (FSC) and side scatter (SSC) parameters; 2) single cells were discriminating plotting the area of the FSC vs the width of FSC; 3) live/dead staining was uses to exclude dead cells from analysis: live cells are negative for the live/dead staining; 4) the different cell populations were identified based on the surface markers expression.

☐ Tick this box to confirm that a figure exemplifying the gating strategy is provided in the Supplementary Information.
