# Supplementary figures and images for: Cardiolipin preserves Treg metabolic fitness and immune homeostasis in the gut
Source: Nat Metab. 2026 May 18;8(6):1368–89. doi: 10.1038/s42255-026-01533-9 (PMC13303090; doi:10.1038/s42255-026-01533-9)

Figure 5c

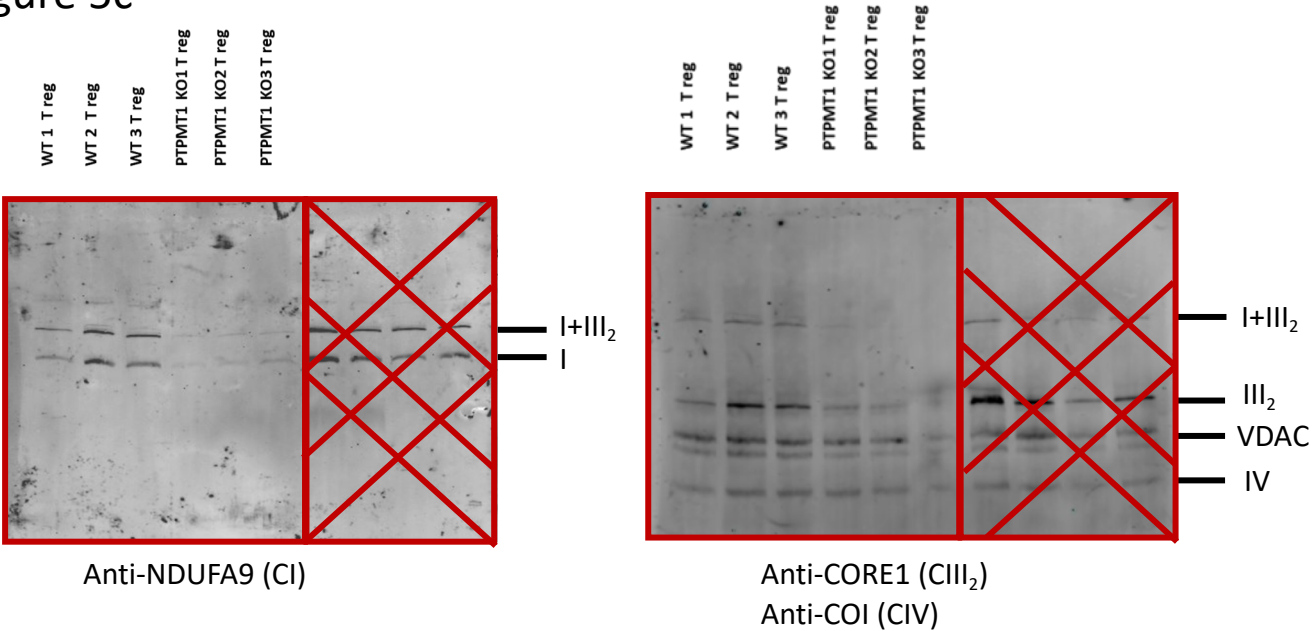

Figure 5d

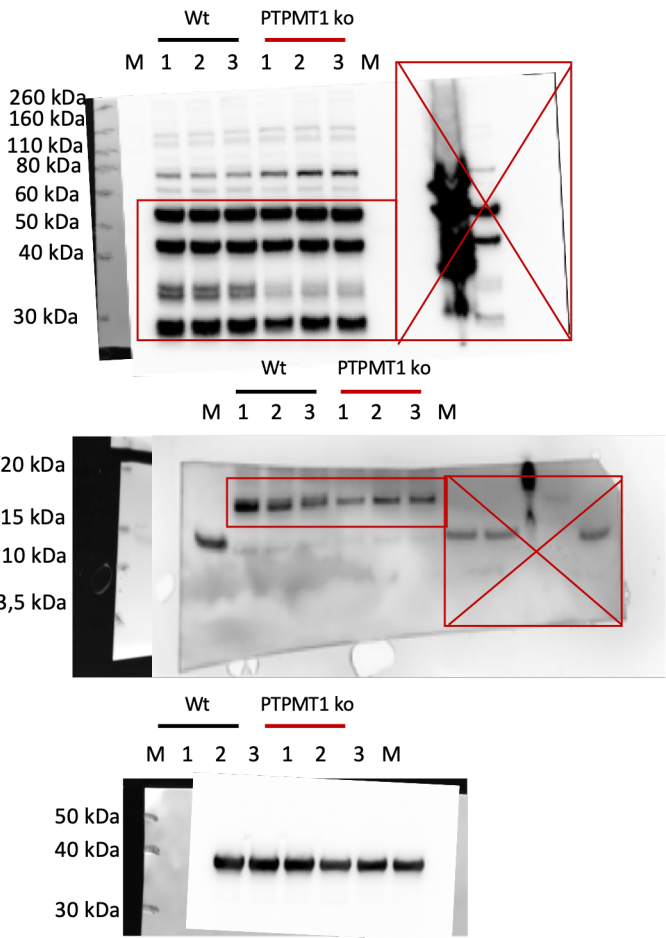

Figure 5l

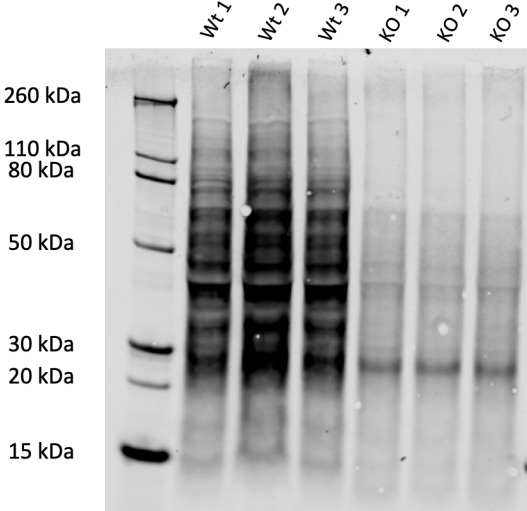

Figure 5m

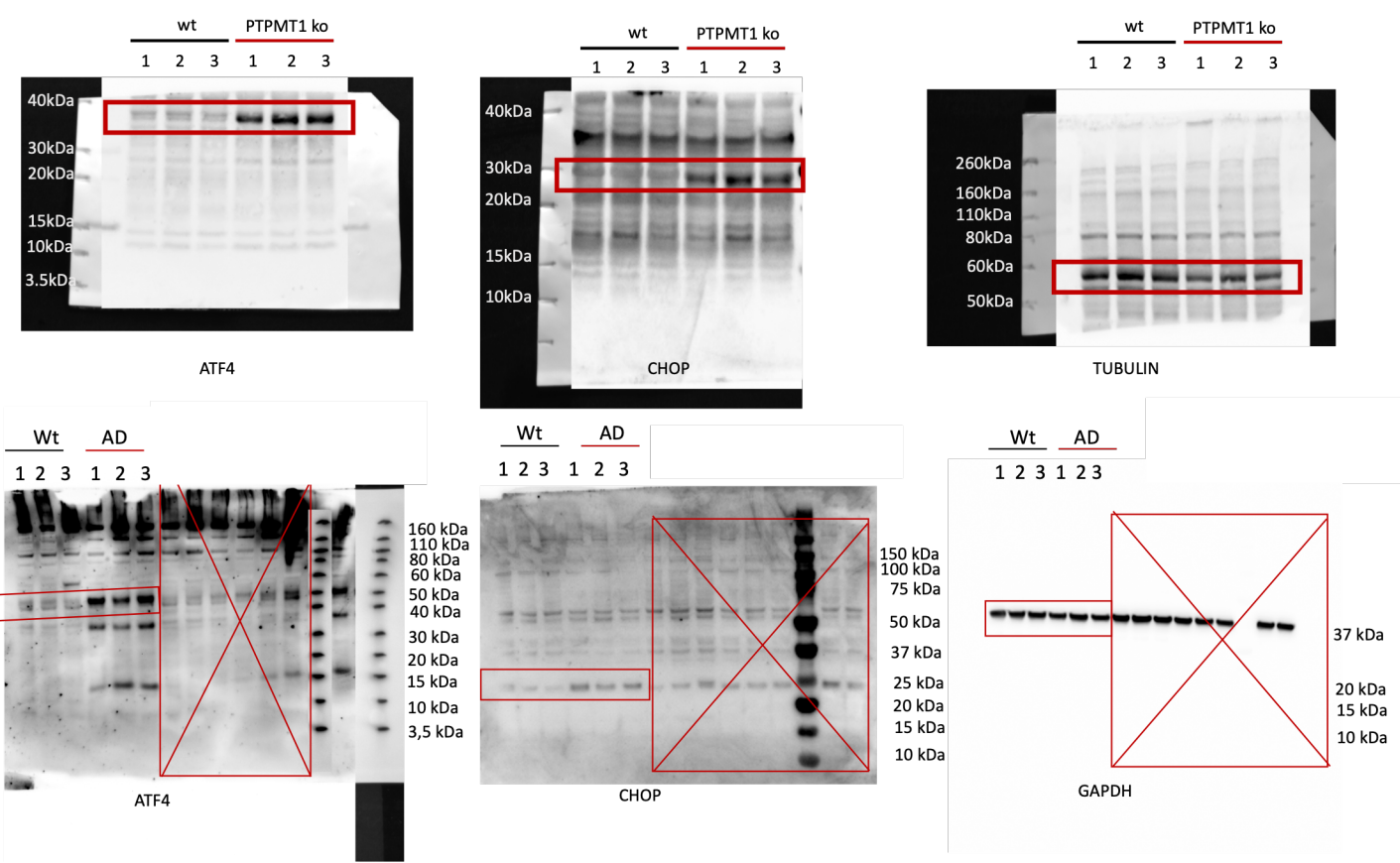

Extended Figure 5c

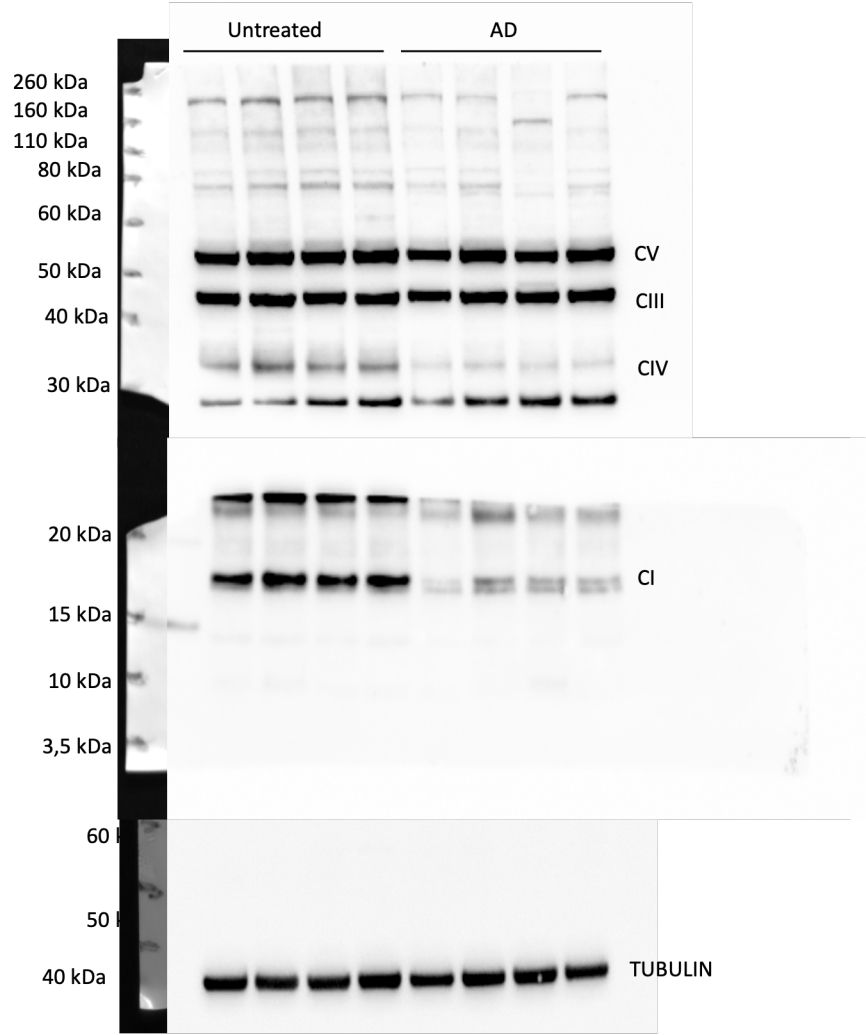

Extended Figure 6b

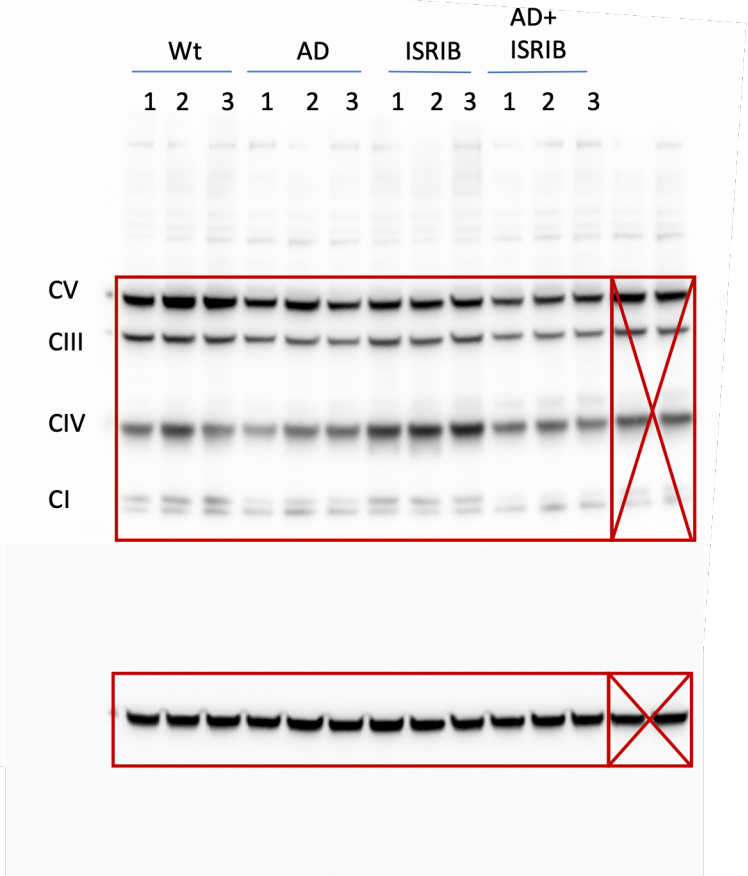

Supplement: Supplementary file 10 — Statistical source data. [file 42255_2026_1533_MOESM10_ESM.pdf]
